# Energy Absorption of Natural-Fiber-Reinforced Composites: Influencing Factors, Mechanisms, and Sustainability Perspectives

**DOI:** 10.3390/ma19204424

**Published:** 2026-10-21

**Authors:** Lei Yao, Taiqu Liu, Zhe Cao

**Affiliations:** College of Mechanical Engineering, Jiangsu Normal University, Xuzhou 221116, China

**Keywords:** natural-fiber-reinforced composites, energy absorption, crashworthiness, impact behavior, global warming potential, sustainable composites

## Abstract

**Highlights:**

- Reviews multiscale factors governing energy absorption in natural-fiber-reinforced composites.
- Introduces an SEA production-related GWP map for performance–sustainability comparison.
- Identifies multiscale heterogeneity, data comparability, and durability as key research gaps.

**Abstract:**

Natural-fiber-reinforced composites (NFRCs) have been shown to be an alternative material for use in impact protection and crashworthiness because of their low density, favorable specific properties, renewable resources, and potential environmental benefits. This paper will cover the mechanisms and key parameters governing the energy-absorption behavior of natural-fiber-reinforced composites, with emphasis on fiber and matrix types, fiber–matrix interfaces, particulate modification, reinforcement architecture, fiber orientation, stacking sequence, fiber volume fraction, environmental conditions, and structural geometry. The available evidence shows that energy is dissipated through matrix cracking and plastic deformation, interfacial debonding and sliding, fiber bridging, pull-out and fracture, crack deflection, delamination, and progressive crushing. Maximizing fiber strength, interfacial adhesion, fiber content, or laminate thickness does not necessarily maximize energy absorption. Instead, optimal performance requires a balance between effective load transfer and stable progressive damage. Moisture can further produce competing effects through short-term plasticization and long-term interfacial degradation. Current research is limited by inconsistent testing protocols, insufficient normalization of energy absorption metrics, incomplete understanding of multiscale damage evolution, and limited long-term and component-scale validation. Future studies should integrate multiscale characterization, validated numerical modeling, standardized testing, and multi-objective optimization to develop lightweight, damage-tolerant, and sustainable energy-absorbing structures.

## 1. Introduction

Due to recent efforts to create a lower carbon footprint and conserve resources more efficiently, engineers have been looking for ways to produce lighter materials [1,2]. Using natural fibers as reinforcements typically consumes lower amounts of non-renewable energy compared to traditional reinforcement. Some of the initial research on life-cycle assessments (LCAs) showed that some NFRCs could use around 37% less energy compared to glass-fiber-reinforced composites (GFRCs) [3]. Replacing 30 wt.% glass fiber with 65 wt.% hemp fiber in a metric ton of thermoplastic was predicted to decrease non-renewable energy consumption by 50,000 MJ and CO_2_ emissions by roughly 3 metric tons [3]. Due to their lower densities and high specific properties, NFRCs have gained interest as lightweight engineering materials. From a broader sustainability perspective, NFRCs also relate to several United Nations Sustainable Development Goals (UNSDGs). Their low density and potential for structural lightweighting can help improve energy efficiency in transportation and services. This is consistent with SDG 7 (Affordable and Clean Energy), especially Target 7.3, which calls for faster progress on energy efficiency. In many systems, renewable lignocellulosic fibers can replace synthetic reinforcements that consume large amounts of energy. These systems also tend to need less energy production and produce fewer greenhouse gas emissions. This aligns with SDG 9 (Industry, Innovation and Infrastructure), particularly Target 9.

The development of automotive, aerospace, transportation, packaging, construction, and protective systems has also increased the demand for lightweight structures capable of dissipating impact and crash energy in a controlled way. Energy absorption governs the ability of a material or structure to limit force transmission, suppress uncontrolled failure, and protect occupants, equipment, or transported products [4]. Metallic energy absorbers offer predictable plastic collapse and mature manufacturing, yet their high density limits further weight reduction. Carbon-fiber-reinforced composites (CFRCs) and GFRCs offer excellent specific stiffness and strength, yet their environmental performance is constrained by the embodied energy of synthetic fibers and recycling difficulties. NFRCs therefore represent a potentially attractive alternative because their energy absorption is governed not only by constituent strength but also by multiple damage mechanisms that dissipate energy [1,5].

**Figure 1:**
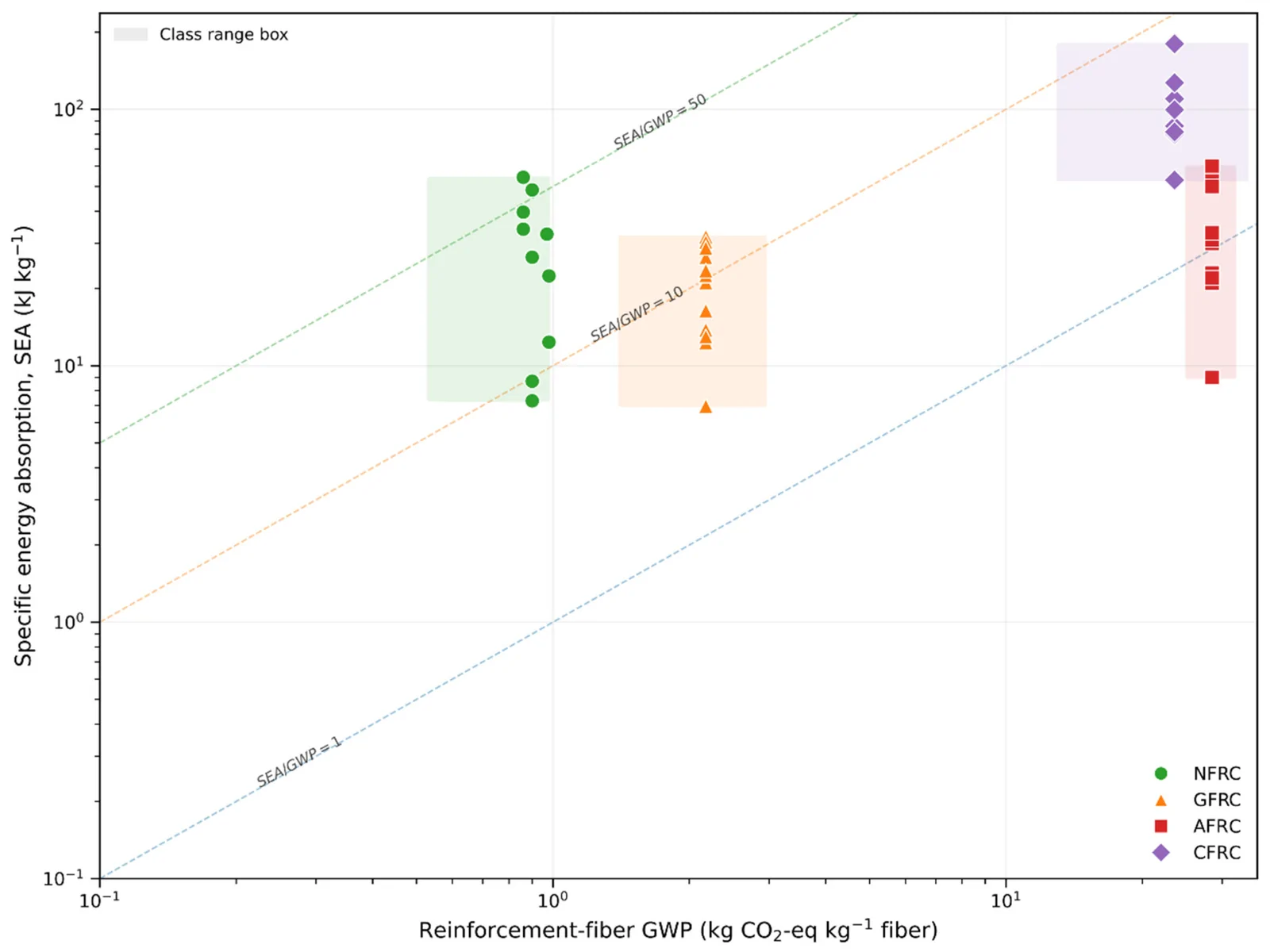
Specific energy absorption (SEA) versus cradle-to-gate global warming potential of reinforcing fibers (GWP*_f_*) for NFRCs, GFRCs, aramid-fiber-reinforced composites (AFRCs), and CFRCs. Scatter points represent reported composite SEA values, while the shaded rounded regions indicate the reported GWP range of the corresponding reinforcing fibers. Dashed lines represent constant SEA/GWP*_f_* indices and are intended as comparative guides for visualizing the performance–environmental trade-off [1,6,7,8,9,10,11,12,13,14,15,16,17,18,19,20,21,22,23,24,25,26,27,28,29,30,31].

NFRCs become more attractive when considering both energy absorption and reinforcement environmental impact. Figure 1 compares reported composite specific energy absorption (SEA) with the cradle-to-gate global warming potential (GWP) of the corresponding reinforcing fibers (*GWP_f_*). NFRCs predominantly occupy the low-*GWP_f_* region while exhibiting *SEA* values that overlap with, and in some cases exceed, those of GFRCs. CFRCs and aramid-fiber-reinforced composites (AFRCs) can achieve higher *SEA* values, but this is generally associated with substantially greater *GWP* associated with the reinforcement.

The shaded regions in Figure 1 represent the reported *SEA* and fiber-*GWP* ranges of the corresponding composite classes, whereas the dashed lines denote constant *SEA*/*GWP_f_* indices.

$$\frac{SEA}{{GWP}_{f}}=C\;(C\;\mathrm{is}\;a\;\mathrm{constant}\;\mathrm{value}.)$$

A higher *C* indicates a higher SEA at the same fiber-GWP level. These lines are intended as comparative guides to show the trade-off between composite-level energy absorption and environmental burden associated with reinforcement. Thus, the main advantage of NFRCs may not be absolute SEA. Instead, it may be a favorable balance between energy absorption and environmental burden associated with the reinforcement.

NFRCs are commonly produced by combining lignocellulosic fibers, including flax, hemp, jute, kenaf, sisal, ramie, coir, and bamboo, with thermosetting or thermoplastic matrices [32]. Their energy absorption response emerges from interacting deformation and damage mechanisms operating across the constituent, interface, laminate, and structural scales [33,34,35]. External work may be dissipated through matrix deformation and cracking, fiber-matrix debonding, frictional sliding, fiber pull-out and fracture, delamination, splaying, local buckling, and progressive crushing [33,34,36,37,38,39,40]. The hierarchical morphology of natural fibers may provide additional deformation pathways through lumen collapse, cell-wall bending, bundle splitting, and progressive compaction [34]. Consequently, high strength or strong interfacial adhesion does not necessarily correspond to maximal energy absorption; effective dissipation often requires a balance between load transfer and controlled damage evolution.

Previous reviews have established an important foundation for understanding the impact response and crashworthiness of NFRCs, but their scope has generally emphasized specific material classes, loading regimes, or structural configurations. Recent reviews of natural fiber and hybrid thin-walled structures have focused on automotive crashworthiness, including cross-sectional geometry, stacking sequence, triggering and filling strategies, loading rate, and axial crushing behavior [37]. Other recent reviews have concentrated on the impact strength of natural-fiber/epoxy composites, particularly the effects of fiber content, fiber length, chemical treatment, stacking sequence, hybridization, manufacturing conditions, and fillers [39]. Theoretical and computational approaches for predicting the impact response of NFRCs have also been reviewed [41]. These studies provide valuable perspectives specific to material, processing, structural, or modeling, but the relationships among characteristics at the constituent scale, interfacial processes, reinforcement architecture, environmental effects, progressive damage, and energy absorption at structural scale are still not well integrated within a common mechanistic framework.

The principal gap is therefore not the absence of studies on individual influencing factors or damage mechanisms but the lack of a consistent framework linking them across structural scales. Fiber type, matrix behavior, interfacial modification, fiber content, reinforcement architecture, moisture exposure, and structural geometry are commonly discussed as separate variables, although their effects on energy absorption are transmitted through a connected sequence of load transfer, damage initiation, crack propagation, interfacial sliding and pull-out, local instability, progressive crushing, and densification. Similar changes in absorbed energy may consequently arise from fundamentally different mechanisms, making apparently contradictory findings difficult to reconcile when the dominant damage process and loading scale are not considered. In addition, material- and laminate-scale impact behavior is frequently treated separately from structural crashworthiness, while environmental performance is rarely evaluated together with energy absorption efficiency. A framework capable of connecting these levels is therefore needed to move the literature from comparisons of individual parameters to interpretations and designs based on mechanisms.

Comparison is further complicated by the diversity of experimental methods and performance indicators. Drop-weight impact, ballistic penetration, quasi-static crushing, and dynamic crash tests impose different stress states and failure mechanisms. At the material and laminate scales, absorbed energy should be interpreted together with force, displacement, damage, and residual properties, whereas structural crashworthiness is more appropriately assessed using SEA, crushing forces, efficiency, and deformation stability. Meaningful cross study comparison requires explicit consideration of loading mode, geometry, rate, boundary conditions, damage state, and the physical meaning of the reported metric.

To address these gaps, this review develops a multiscale framework centered on mechanisms that links experimentally controllable design variables to damage evolution and energy dissipation from the constituent level to the structural level. At the nano- and microscale, cell-wall organization, microfibrillar structure, constituent properties, and fiber-matrix interactions govern local deformation, load transfer, and damage initiation. At the mesoscale, fiber bundles, interfaces, pores, gradients, and laminate architecture regulate debonding, frictional sliding, fiber bridging and pull-out, crack deflection, delamination, and stress redistribution. At the macroscale, these processes collectively determine local buckling, progressive crushing, collapse stability, densification, and the resulting energy absorption metrics. The novelty of the proposed framework therefore lies not in introducing unknown individual damage mechanisms but in integrating established mechanisms across structural scales and explicitly linking them to fiber type, matrix selection, interfacial modification, reinforcement architecture, environmental exposure, and structural geometry. This approach also bridges material- and laminate-scale impact behavior with structural crashworthiness, enabling apparently inconsistent findings to be interpreted through differences in dominant damage mechanisms and test conditions. Beyond the mechanical perspective, this review assembles a quantitative SEA-GWP database for natural-fiber, hybrid natural-fiber, glass-fiber, carbon-fiber, aramid-fiber composite, and metallic energy-absorbing systems to examine performance–sustainability trade-offs within a common framework. Finally, current limitations and future priorities are assessed in terms of standardized testing, multiscale characterization, environmental durability, predictive modeling, technological maturity, and sustainable engineering implementation.

## 2. Experimental Methods and Performance Evaluation

NFRCs can be subjected to many forms of loading to assess their energy absorption properties. Quasi-static, low-velocity impact, and high-strain-rate loading have all been used. The properties that are obtained are highly dependent on the shape and configuration of the test specimen, loading rates, and reduction in the data used. Table 1 lists some of these testing techniques, specimen shapes, and values obtained from each, along with the references that discuss each technique.

### 2.1. Quasi-Static Compression

Tests for quasi static compression and axial crushing are commonly performed to determine the compressive strength, failure pattern, and energy absorbing properties of NFRCs [43]. Depending on what the specimen consists of, different geometries will be used. Usually, the plate compression specimens are made following the guidelines in ISO 14126 and ASTM D6641 [44,45]. Tubes are commonly used when looking into crashworthiness, and these usually consist of round or polygonal shapes that are crushed between two plates in the axial direction. The outer diameter of these tubes can vary but typically ranges from 20 to 50 mm, with a length-to-diameter ratio of around 2–3. These dimensions help prevent overall buckling and allow for more progressive crushing [46,47,48,49,50,51]. It is important that the tube is properly aligned axially, as this can affect the crushing pattern and amount of energy absorbed.

Quasi-static tests are usually conducted under displacement control. Loading rates of 0.1–10 mm/min are common, corresponding to nominal strain rates of roughly 10^−4^ to 10^−2^ s^−1^; these rates help minimize dynamic effects [52,53]. The appropriate rate depends on specimen geometry and material configuration. Comparisons across study should account for the loading rate. Nominally quasi-static data should not be treated as directly equivalent.

**Figure 2:**
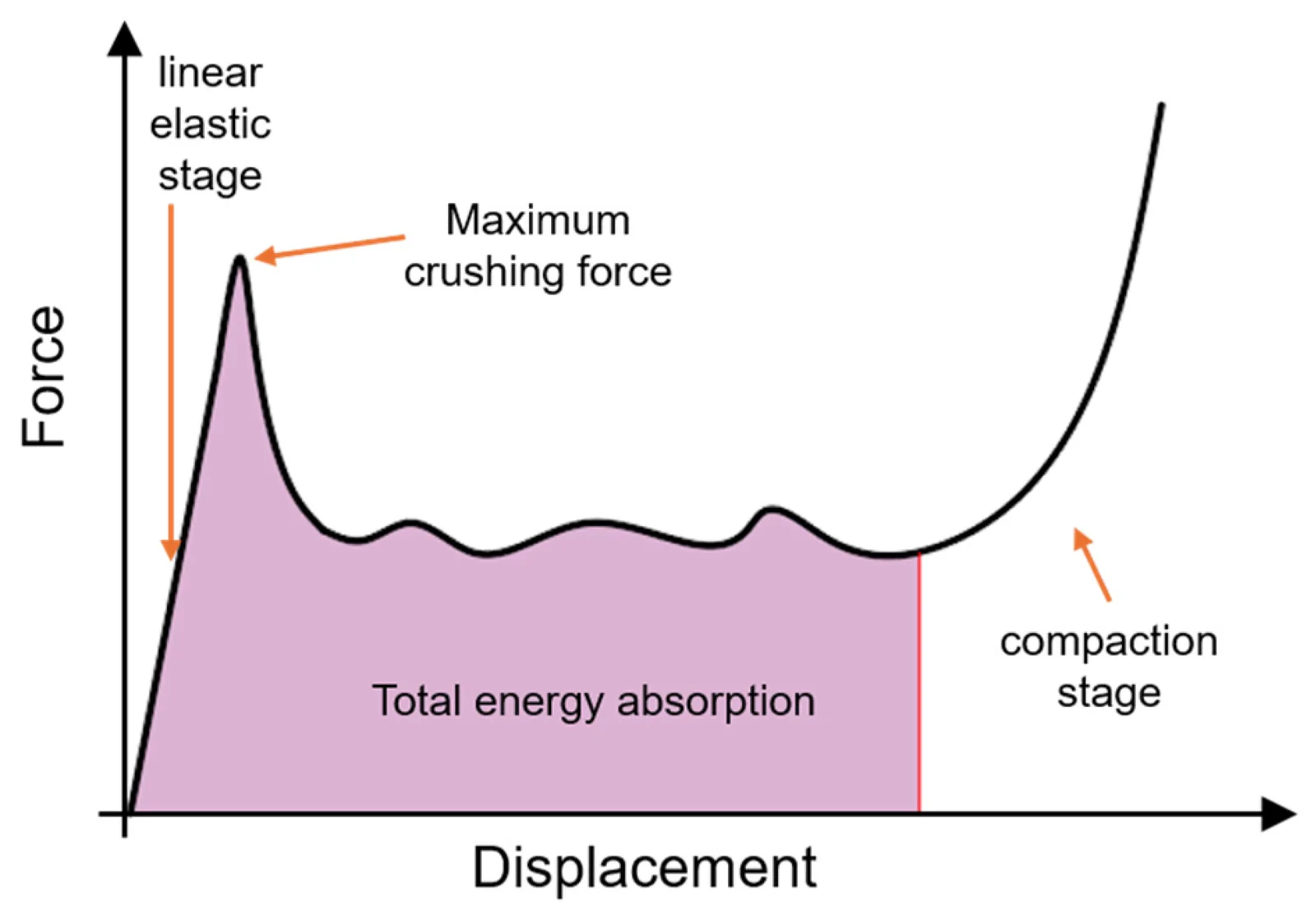
Typical force–displacement response curve under quasi-static compression.

The force-displacement curve is the main basis for interpreting the crushing response. A typical response includes initial elastic loading, damage initiation associated with the peak load, a progressive crushing plateau, and final densification [51,54]; the plateau region is especially important. Stable progressive crushing generally indicates sustained energy dissipation through matrix cracking, fiber fracture, interfacial debonding, delamination, friction, and local folding. Therefore, evaluation should consider not only the peak load but also how stable the post-peak response is. A typical force-displacement response curve, as shown in Figure 2, can be divided into four stages: the linear elastic stage, the post-peak decline stage, the plateau stage, and the compaction stage [51].

The main crashworthiness parameters are derived directly from the force–displacement curve. Total absorbed energy is

$$EA=\int_{0}^{\delta }F\left(\delta \right)d\delta$$

and specific energy absorption (SEA) is

$$SEA=\frac{EA}{m}$$

where *m* is the mass of the sample. The maximum crushing force (MCF) corresponds to the peak force, whereas the mean or plateau crushing force can be expressed as follows:

$$PCF=\frac{1}{\delta_{2}-\delta_{1}}\int_{\delta_{1}}^{\delta_{2}}F\left(\delta \right)d\delta$$

Crushing efficiency (CFE) is commonly defined as follows:

$$CFE=\frac{PCF}{MCF}\times 100\%$$

SEA is especially useful for comparing lightweight structures, while CFE reflects how stable load transmission is during progressive collapse. Even so, these metrics should not be treated as standalone material properties; they should be interpreted alongside failure mode, specimen geometry, and crushing displacement.

### 2.2. Low-Speed Impact Test

Low-velocity impact testing is widely used to evaluate the impact resistance, damage tolerance, and energy dissipation of NFRC laminates and sandwich structures. These loading conditions represent accidental drops or low-speed contact events. Drop-weight impact is the most commonly reported setup; in this configuration, an instrumented impactor delivers a prescribed incident energy to a supported specimen [55,56,57,58]. Test parameters vary considerably across studies, including impactor mass, impact energy, nose geometry, specimen dimensions, and boundary conditions; they should therefore be reported explicitly.

**Figure 3:**
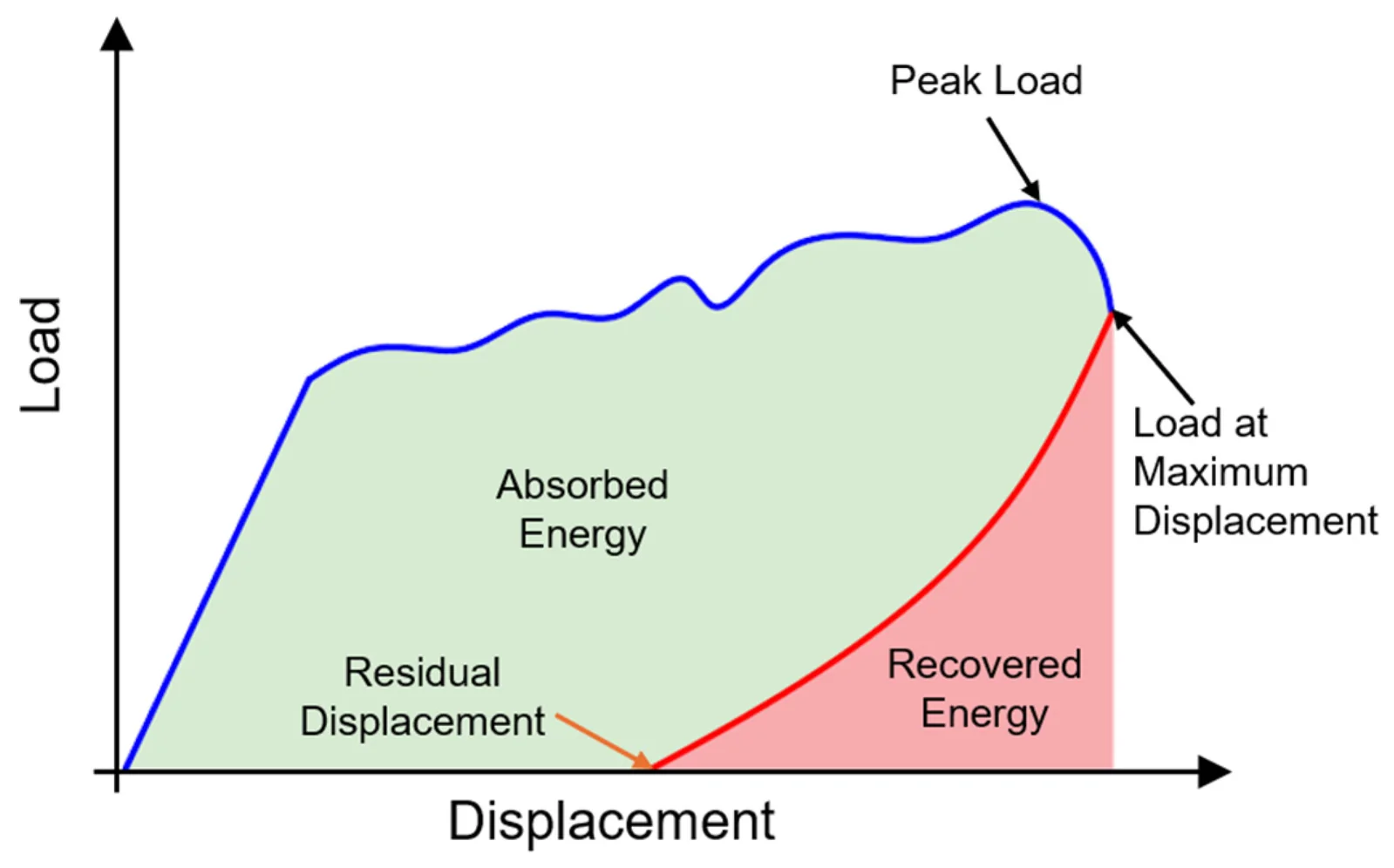
Typical force-displacement response curve under impact conditions.

The variation characteristics of typical force-time curves are shown in Figure 3. Impact response is mainly interpreted from force-time, force-displacement, and energy-time histories. Peak force reflects the initial load-bearing capability; the post-peak response, in turn, provides information on damage progression. A gradual reduction in force generally indicates progressive matrix cracking, delamination, fiber-matrix debonding, and fiber fracture. An abrupt post-peak drop is more commonly associated with brittle failure or behavior governed mainly by penetration [38,59,60,61,67,68,69,70,71,72,73].

The area under the force-displacement curve represents the mechanical energy absorbed during the impact event. The final value of the energy versus time curve gives an additional estimate of absorbed energy after the impactor and specimen separate [74,75]. Even so, high absorbed energy should not automatically be interpreted as superior impact resistance-extensive damage or perforation can also increase energy dissipation. Impact performance need to assess together with peak force, rebound behavior, displacement, damage area, and residual mechanical properties [76]. Comparative studies further show that matrix ductility and interfacial characteristics can substantially modify the impact response. For example, flexible jute/rubber systems showed greater energy absorption than corresponding stiff epoxy based systems in the reported configurations [77,78].

### 2.3. High-Strain-Rate Impact Test

Tests that are able to measure the response of NFRC at high strain rates are necessary. Impacts, blasts, and crashes are all examples of events where materials experience high strain rates. One common method that can be used to measure dynamic compression of a material is the Split-Hopkinson Pressure Bar (SHPB) [64,79,80]. By measuring incidents, reflected and transmitted stress waves, we can calculate the dynamic stress-strain curve. The assumption of one dimensional wave propagation is made. To obtain reliable data, several factors must be considered: a uniform stress distribution throughout the specimen, a known strain rate, adequate contact between the specimen and bar, and accurate strain gauge readings. Techniques such as pulse shaping and reducing friction are used to help achieve these goals [65,66,81,82].

There are many papers that have shown NFRCs to be strain-rate-dependent. When flax fiber composite was loaded at strain rates as high as 2200 s^−1^, the response changed as the strain rate increased, from fiber-matrix debonding to fiber pull-out and fiber fracture [80]. Bamboo fiber/epoxy and sisal reinforced composites also have shown strain-rate-dependent responses when compressed, as their strength, deformation, and energy absorption capabilities all changed with increasing strain rate [65,83,84]. It can be seen that material behavior is determined not only by strain rate but also by the type of fibers used and their volume fraction, configuration, and the behavior of the matrix.

More research needs to be carried out in order to compare the results of different experiments. Natural fiber heterogeneity causes substantial experimental scatter. In addition, specimen dimensions, strain-rate windows, end friction, stress equilibrium conditions, and signal processing vary across test configurations [62,66,79,85]. Environmental conditions further complicate interpretation because temperature and moisture can modify matrix properties and interfacial behavior [86]. High-speed imaging, digital image correlation, pulse shaping, and full-field measurements are therefore increasingly used to improve the reliability of dynamic measurements and clarify damage evolution [42]. More consistent specimen preparation, reporting procedures, and dynamic testing protocols are still needed to enable quantitative comparison across different NFRC systems.

Direct comparison of energy absorption values obtained from different test methods requires caution because the measured response depends on both the material and the test configuration. Specimen geometry influences stiffness, deformation mode, crushing stroke, and failure localization; changes in laminate thickness, span, tube diameter-to-thickness ratio, length-to-diameter ratio, or triggering geometry can therefore alter both EA and SEA even for nominally identical material systems. Loading rate introduces additional differences because NFRCs are sensitive to strain rate. Increasing the loading rate can modify the matrix response, fiber-matrix stress transfer, peak load, inertia effects, and the relative contributions of cracking, debonding, pull-out, and fracture. It should be noted that high-rate tests can produce different strength and energy responses from quasi-static loading.

Boundary conditions also determine the stress state and accessible damage mechanisms. Axial crushing between rigid platens favors progressive collapse, whereas supported or clamped plates under drop-weight impact develop combinations of bending, indentation, matrix cracking, delamination, and penetration. End friction, eccentricity, support span, and clamping stiffness can further modify the measured response. Finally, the testing method and energy definition must be considered. Quasi-static EA is generally obtained by integrating the force-displacement curve over a prescribed crushing displacement, whereas drop-weight impact energy depends on incident, rebound, and absorbed impact energy, and SHPB measurements are derived from stress-wave signals under dynamic equilibrium assumptions. Consequently, normalization by specimen mass to obtain SEA improves comparison based on mass but does not eliminate differences arising from geometry, loading rate, boundary conditions, or failure mode. Cross-study comparisons should therefore be carried out preferentially within comparable testing regimes and interpreted as engineering trends.

## 3. Energy-Absorption Mechanism of NFRCs

**Figure 4:**
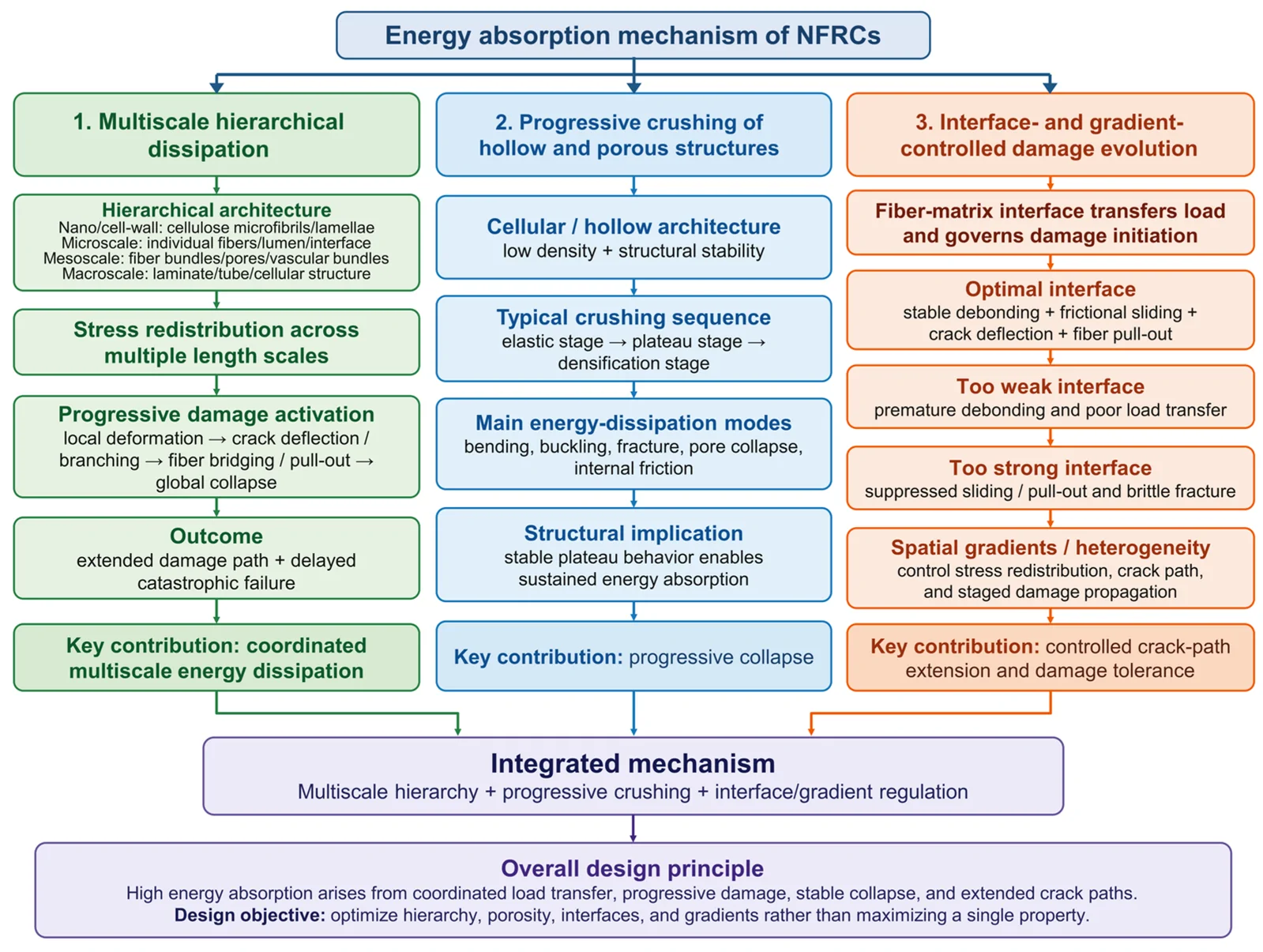
Multiscale framework for the energy-absorption mechanisms of NFRCs.

The energy absorption of NFRCs does not depend only on the strength and stiffness of their components. It is a function of hierarchical architecture, cells, interfaces, and heterogeneities throughout the structure. There are many levels of hierarchy within natural fibers and plants, encompassing the cell wall, lumen, fiber, vascular bundles, pores, and the shape of the structure on a larger scale [87,88,89,90,91,92]. Each level has its own deformation and damage modes, which are activated under compressive or impact loading. These modes allow the applied force to be dissipated over a greater time and distance before complete failure occurs. Therefore, we can describe some key energy absorption mechanisms of NFRCs, including multiscale hierarchical dissipation, progressive cell crushing, and interface/gradient effects. Energy absorption mechanisms of NFRCs are shown in Figure 4.

### 3.1. Multiscale Hierarchical Structure and Energy Dissipation

Hierarchical structures allow for deformation and damage to propagate through the various scales; their architecture spans from nanoscale cellulose constituents and layered cell walls to microscale fibrous units, mesoscale pores and vascular bundles, and macroscopic structural geometries [93,94,95]. At smaller scales, deformation resistance and crack initiation are influenced by cell-wall composition, lamellar organization, microfibril arrangement, and local geometry [96,97,98]. At larger scales, fiber bundles, pores, and heterogeneous structural regions redistribute stresses and change how cracks propagate afterward.

Under compression or impact, damage can thus evolve progressively from local deformation to larger-scale structural collapse. It does not immediately concentrate on a single critical site [34,99,100]. Microscale wall deformation and interfacial motion may be followed by mesoscale crack deflection, bundle separation, and fiber bridging. Eventually, macroscopic crushing and compaction take over. This sequential activation increases the number of dissipative processes involved and extends the damage-development path.

Bamboo is a good example of this multiscale interaction. Its heterogeneous distribution of vascular bundles, parenchyma, vessels, and pores controls local stiffness and crack trajectories. Mechanisms such as interfacial debonding, fiber bridging, pull-out, and crack deflection further delay fracture [101,102,103]. Thus, the energy absorption capability of hierarchical natural systems comes from coordinated deformation and damage across structural scales. It does not come from a single high strength constituent [38,104,105].

### 3.2. Progressive Crushing of Hollow, Porous, and Multicellular Structures

As discussed earlier, hollow and porous structures can also exhibit low mass and prolonged energy absorption. Wood, bamboo, and pomelo skin, along with other multicellular structures, exhibit a response to compression that is significantly influenced by their relative density and the distribution and scale of their pores [106,107,108]. Typically, under compression, they will show elastic behavior at first, then transition to a plateau as they fail, and then eventually become fully dense. During the failure plateau, there is repeated flexing and failure of pores or cells, allowing for prolonged energy absorption [109].

The efficiency of this mechanism depends not only on the porosity but also on the cellular architecture. In wood, it has been found that specific energy absorption is highly dependent on the amount of meso-pores and macro-pores. Therefore, structures with similar porosity will exhibit different crushing behaviors [110], similar to what we see in nature with bio-inspired cellular structures. These structures have multiple layers, which will cause crack propagation between layers, layer contact, and buckling or densification, thereby delaying structural failure [111]. Cellular structures that are graded will also begin to deform in weaker areas and propagate the deformation to stronger areas, causing controlled failure of the structure [112].

These findings indicate that effective porous energy absorbers require control over the sequence of collapse, not simply maximization of porosity. Stable progressive deformation, delayed densification, and avoidance of premature global buckling are therefore central structural requirements for achieving high energy absorption at low mass.

### 3.3. Interface- and Gradient-Controlled Damage Evolution

Another aspect of energy absorption in NFRCs is through the interface, which allows for load transfer as well as providing methods for damage to dissipate energy. There are many forms of damage that occur during impact or fracture of the material, such as matrix cracking, interface debonding, friction from sliding, fiber pull-out, fiber breaking and crack deflection [113,114,115]. Depending on how the interface is bonded, there will be more or less of each damage process. If the interface is too weak, the interface will not allow for proper load transfer and can result in too much interface debonding and pull-out. However, if the interface is too strong, there will be little to no sliding at the interface, and fiber failure will occur. Thus, a good balance needs to be found in order to maximize energy absorption. The interface must be able to transfer loads to the fibers but allow some debonding, sliding, and pull-out [116,117].

Additional factors can also influence the interface and its ability to absorb energy. Moisture absorption, for instance, can cause the material to swell. This can result in non-uniform deformation throughout the material, giving rise to stress, cracking, and a reduction in shear strength at the interface [101,118,119]. All of these will have an effect on how much load transfer occurs and how much damage occurs through interface debonding.

Gradients also play a role in controlling the failure process. Many natural materials have variations in amount of fiber, porosity, tissue density, stiffness, and strength throughout the cross-section of the material [120]. If these gradients are created properly, they allow stiffer areas to withstand the applied load while allowing more compliant areas to fail, allowing for a lower stress concentration at any one point, and controlling where and how damage will propagate through the material [121]. Bamboo and other hierarchically structured materials can take advantage of gradients through the radius. They also have multiple vascular bundles and directions. These features cause crack deflection, crack bifurcation, crack bridging, bundle pull-out, and sliding along interfaces [103,122,123,124,125]. By engineering these interfaces and using gradients, we can cause damage to propagate through our material in a more gradual fashion. Thus, from an energy absorption standpoint, we want to control how the load will travel through the material and how it will fail and densify.

Studies have shown that the ability of a fiber/matrix system to absorb energy through debonding, pull-out, and delamination is dependent on the interface and ductility of the matrix material. The use of cellulose acetate to toughen the interface in a flax/epoxy laminate was able to increase the perforation threshold by 66% and perforation energy by 42%. It was also noted that there was an increase in debonding and more progressive failure [126]. This suggests that controlled debonding can enhance impact tolerance when sufficient load transfer is maintained.

Table 2 summarizes representative quantitative evidence linking interfacial modification, damage evolution, and energy-absorption response in NFRCs. Graphene-oxide (GO) modification provides a complementary example. In GO-modified flax/epoxy composites, interfacial adhesion increased by approximately 40% and void content decreased from 2.41% to below 0.04%, while perforation energy changed only slightly [126,127]. In another flax/epoxy/GO system, crack initiation energy increased from 3.80 to 6.10 J, whereas crack propagation energy decreased from 15.10 to 11.90 J [128]. This suggests that interface strengthening can redistribute energy from subsequent damage propagation toward delayed damage initiation. Matrix ductility also modifies the role of delamination. Compared with flax/epoxy, flax/MAPP composites exhibited more than 50% higher perforation energy while showing a 38–59% smaller damaged area and limited delamination growth [129]. Thus, extensive delamination is not a prerequisite for high energy absorption. These results support an optimal interface concept in which load transfer is balanced with controlled debonding, sliding, pull-out, and limited delamination.

## 4. Multiscale Factors Governing Energy Absorption

### 4.1. Constituent-Level Factors

#### 4.1.1. Fiber Type

When it comes to the impact and energy absorption properties of NFRCs, one of the factors that will play a role is fiber type. Fibers vary greatly in their tensile strength, elastic modulus, strain at failure, tensile toughness, density, lumen shape, microfibril angle, chemistry, surface roughness, and amount of flaws. These intrinsic characteristics affect not only the load-bearing capacity of the reinforcement but also the competition among fiber deformation, fiber fracture, interfacial debonding, frictional sliding, and fiber pull-out during impact. Consequently, the incorporation of fibrous reinforcement does not necessarily improve the impact performance of a polymer matrix, and no universal ranking of natural fibers can be established independently of the matrix, interface, reinforcement architecture, fiber content, and loading conditions [59,130,131]. A representative example is provided by hemp-fiber-reinforced PLA composites. The impact strength of hemp/PLA composites was approximately 15% lower than that of neat PLA [130]. This reduction was associated with the relatively limited failure strain of hemp fibers and the initiation of cracks at local stress concentrators, such as fiber ends, fiber bundles, incompletely impregnated regions, voids, and discontinuous fiber-matrix interfaces. Under impact loading, these defects may initiate premature matrix cracking and interfacial separation before the reinforcing fibers can make full use of their load-bearing capacity [38,132].

**Figure 5:**
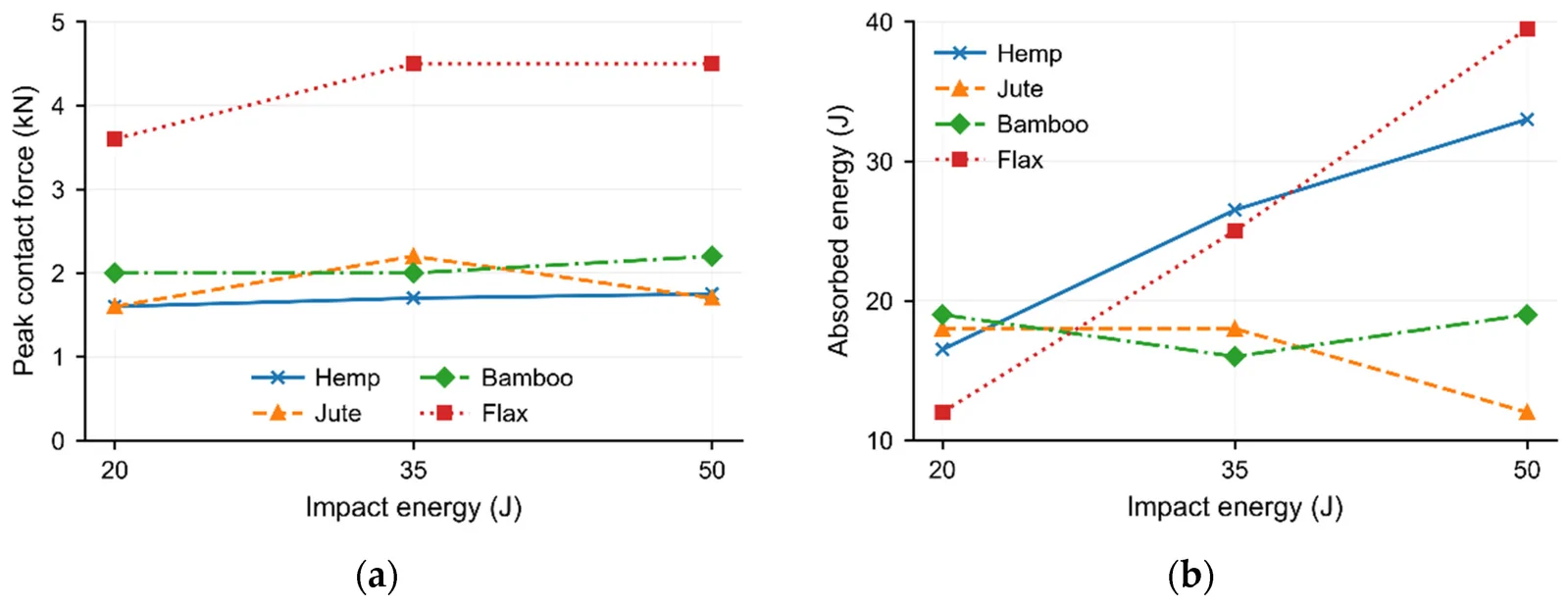
Comparison of (**a**) peak contact forces and (**b**) absorbed energies for different kinds of reinforcement type under 20 J, 35 J, and 50 J (data were sourced from Ref. [59]).

Among the different NFRCs systems shown in Figure 5, flax reinforced composites exhibited the highest peak contact forces, the most stable response, and the best resistance to penetration [59]. At low impact energies, damage was dominated by matrix cracking and delamination, whereas increasing impact energy promoted fiber fracture, bundle separation, penetration, and eventual perforation [99]. These differences may result from the combined effects of fiber type and textile laminate structure. This is especially true when differences in fabric areal density, the thickness of a single ply, and the number of plies required to reach the same nominal laminate thickness are considered [133,134]. Some NFRCs dissipated energy at lower stress levels and higher strains than comparable GFRCs [135]. This low stress, large deformation response may be advantageous in impact mitigation components because it can limit the peak load transmitted to the protected object while maintaining progressive energy dissipation.

Since coir, palmyra palm, and wool have different mechanical properties than normal bast fibers, they can offer more forms of reinforcement in NFRCs. Coir has a lower modulus of elasticity than flax or hemp fibers but has higher elongation, allowing for impact energy absorption due to fiber elongation, pull-out, sliding at the interface and cracking of the matrix. It has been shown in many reviews that the impact performance of coir-reinforced epoxy composites is affected by fiber volume fraction, fiber length, and treatments and processing methods. There is an optimal fiber loading where the impact strength is increased and then decreases due to poor fiber wetting, clumping, and poor fiber interface [39]. Thus, coir’s main advantage is not simply high impact strength but its ability to sustain distributed deformation when the interface and processing quality are controlled. Palmyra palm fibers show a different balance between load-bearing capability and toughness. Hybridization with sisal improves the mechanical response of coir/epoxy systems [136]. Palmyra palm leaf stalk/sisal hybrids with 20 vol.% fiber had impact strengths 24.93% and 10.03% higher than those with 10 and 15 vol.%, respectively; treated hybrids also increased tensile strength by around 62.24% and impact energy by 22.48% relative to the untreated system [137]. Palmyra-based systems therefore benefit particularly from hybrid architecture and controlled interface modification. Wool is a keratin-based protein fiber with high flexibility and extensibility, so its energy absorption depends strongly on architecture and fiber orientation. Abdulla and Hussain reported that wool/polyester composite pipes with a 0°/0° lay-up absorbed approximately 698 and 998 J at 23% and 46% fiber volume fractions, whereas 0°/45° configurations absorbed only 590 and 722 J, respectively [138]. Wool composites can benefit from surface modification, but treatment must be carefully controlled because keratin is sensitive to aggressive alkalis; stronger alkalis degraded wool, whereas mild alkalis and cold-plasma modification gave more favorable surface and mechanical responses [139]. Compared with conventional bast-fiber systems, these fibers represent different energy-dissipation strategies. Flax and hemp suit high stiffness and load-bearing applications, coir offers greater deformability and pull-out/friction dissipation. Palmyra is intermediate and enhanced by hybridization and treatment, and wool offers flexibility but requires orientation control and modification with compatible proteins.

There is also a fiber-type effect when looking at compressive properties of structures. Bambach compared trough-shaped epoxy composites that were reinforced with flax, jute, hemp, glass, and carbon fibers, all with the same fiber mass [140]. The compressive performance of the natural-fiber composites was generally comparable to that of glass-fiber composites. In terms of ultimate load, jute- and flax-reinforced components reached levels similar to those of glass-fiber composites, whereas hemp showed slightly lower values. Hemp nevertheless exhibited a higher buckling load than glass fiber, while jute provided the highest strength efficiency among the investigated natural fibers, followed by flax, which performed at a level comparable to glass fiber [140]. These results demonstrate that some natural fibers can provide competitive structural efficiency in compression-dominated applications. Similar conclusions have been reported for tubular crash absorbers. Flax-fiber-reinforced polymer tubes possess the potential to replace basalt- and E-glass-fiber composites in quasi-static axial crushing applications [9]. This comparison is particularly relevant to crashworthiness because the suitability of reinforcement is determined not only by its peak strength but also by its ability to sustain stable progressive crushing and dissipate energy over a sufficiently long deformation distance. Flax therefore represents a promising reinforcement for lightweight energy-absorbing structures where a balance between structural efficiency, energy absorption, and material sustainability is required. Natural-fiber composites can exhibit fiber-dominated failure with limited delamination, whereas synthetic-fiber laminates may dissipate a larger fraction of the impact energy through delamination [141].

The principal mechanisms through which fiber type affects energy absorption can therefore be summarized as follows: Fibers with high tensile strength and modulus increase load transfer efficiency, contact stiffness, and resistance to initial deformation [57]. Fibers with high failure strain and tensile toughness can accommodate greater deformation and absorb more energy before rupture [142]. Fiber morphology, lumen structure, bundle size, and surface roughness influence stress concentration, crack initiation, mechanical interlocking, and pull-out resistance [34]. Natural defects, kink bands, and variability in technical fibers may cause premature fracture and increase the scatter of the impact results [98,143].

#### 4.1.2. Matrix Type

The matrix used will determine how the NFRC will behave under impact and how much energy it can absorb. By varying the stress transfer between the matrix and fibers, transverse strength, interlaminar strength, ability to yield and propagate cracks, and how the damage progresses can all be altered. This will dictate whether the energy will be absorbed mainly from brittle matrix cracking and delamination or from matrix plastic deformation, interfacial failure, friction, and progressive failure of the structure.

Thermoplastics have more ductile behavior and higher failure strains compared to normal thermoset matrices. They can absorb more energy from matrix yield, viscous plastic deformation, and high laminate deflections [144]. Their ductility may also delay crack initiation and restrict the rapid propagation of matrix cracks and delamination. Thermosetting matrices, particularly conventional epoxy resins, usually provide higher stiffness, strength, dimensional stability, and fiber-matrix adhesion, but their highly crosslinked molecular networks limit plastic deformation and can promote brittle matrix cracking under impact [129]. Nevertheless, thermoplastic composites should not be considered universally superior because their lower stiffness or weaker interfacial bonding may reduce the peak load, structural stability, and post-impact load-bearing capacity [69]. The specific energy absorption of PLA-matrix-based NFRCs is significantly higher than that of HDPE (high-density polyethylene) [145]. This can be attributed to the higher modulus and strength of PLA, which effectively confines the fibers, prevents local buckling, and enables the structure to absorb energy through stable laminate bending. In contrast, HDPE exhibits insufficient stiffness, leading to easy fiber buckling and resulting in energy absorption solely through inefficient progressive folding.

**Figure 6:**
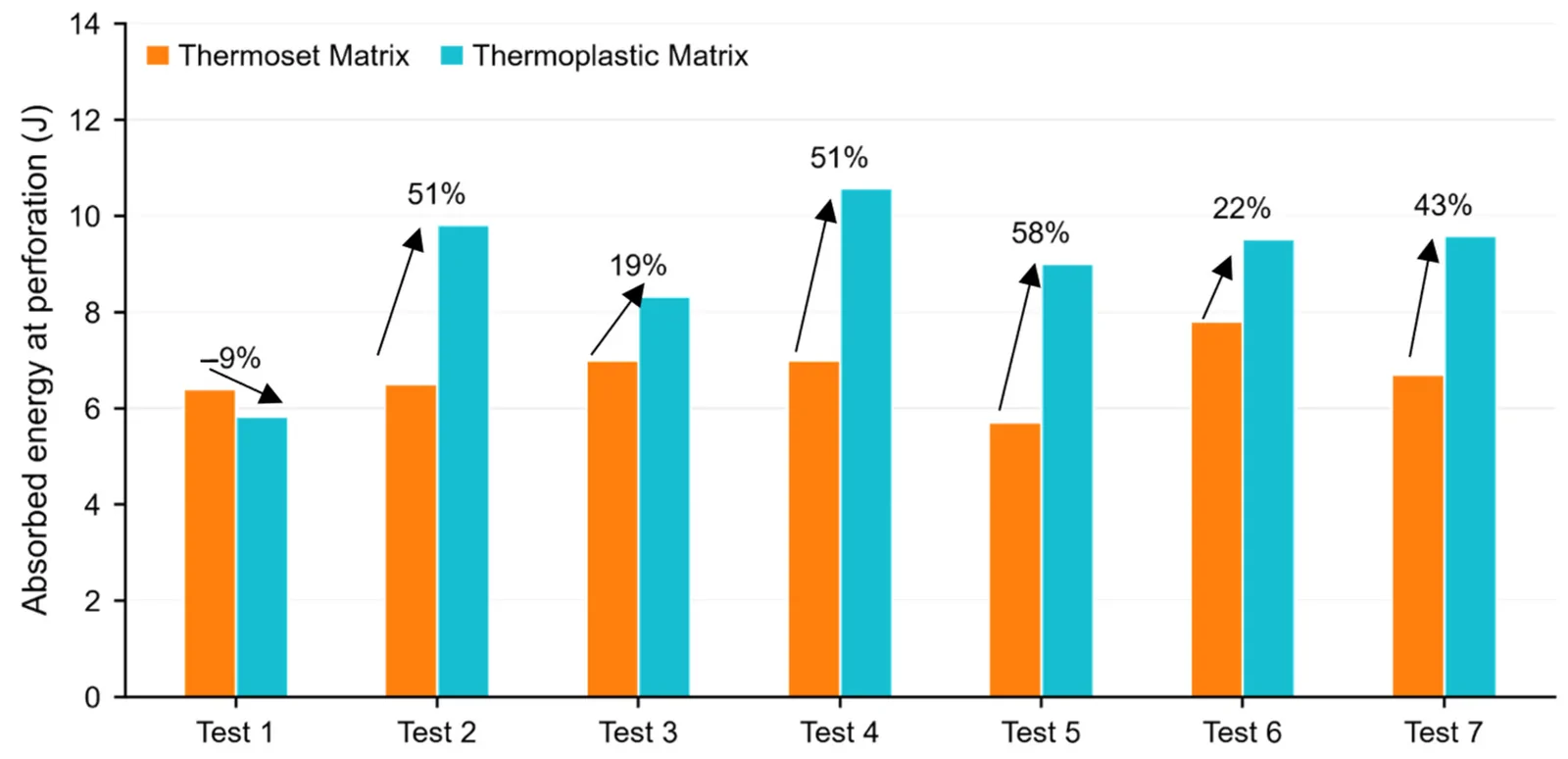
Absorbed energy at perforation of flax/epoxy and flax/MAPP composites (data were sourced from Ref. [129]).

Using a maleic anhydride polypropylene (MAPP) matrix allows flax fiber composites to have greater energy absorption by more than 50% when compared to epoxy, as shown in Figure 6. This is due to MAPP’s ability to dissipate energy through its large amount of elongation at break. Due to it being a tougher matrix, it will reduce crack and delamination growth [129]. It was found that when using flax/basalt hybrid composites that the unmodified polypropylene system had greater perforation energies than both epoxy and compatibilized polypropylene systems [146]. This leads to the conclusion that having better interfacial bonding may decrease interfacial sliding and fiber pull-out.

On the whole, it is best to use thermoplastics as a matrix material if one wants the composite to exhibit high deformations and be able to plastically dissipate energy. Thermoset matrices are preferable for high stiffness and maximum load values as well as better residual properties after impact. The ideal matrix for a crashworthy application would need to have enough stiffness to prevent fiber micro buckling and be tough enough to not fail in a brittle manner [147]. There are many factors to take into consideration when evaluating a matrix, such as maximum force, energy absorption, SEA, deformation, damage area, perforation energy, crush stability, and residual strength after impact [69].

#### 4.1.3. Interface and Particulate Modification

The fiber-matrix interface plays a central role in the mechanical response and energy-absorption behavior of NFRCs. Adequate interfacial shear strength is required to transfer loads efficiently from the relatively compliant matrix to the reinforcing fibers, particularly at high fiber contents, where poor wetting and fiber agglomeration can increase the probability of interfacial defects [142]. Strong interfacial bonding generally improves stiffness, compressive strength, and resistance to damage initiation. However, maximal interfacial strength does not necessarily produce optimal impact performance because impact energy dissipation also depends on controlled debonding, interfacial sliding, and frictional fiber pull-out. The effects of interfacial modification and particulate fillers on the energy-absorption behavior of NFRCs are presented in Table 3.

One of the common methods to treat natural-fiber surfaces is alkali treatment using sodium hydroxide (NaOH). By using the correct concentration and time period, the NaOH solution will dissolve away waxy material on the surface and some of the pectin, hemicellulose, and lignin that surrounds the cellulosic structure. This causes fibrillation of the fibers, increasing the surface roughness and providing more surface area for the fibers, allowing better wetting and mechanical locking between the matrix [161,162]. These changes can enhance stress transfer efficiency and have produced increases exceeding 30% in compressive or related mechanical properties in some specific natural-fiber composite systems [150]. The treatment response depends strongly on fiber species, NaOH concentration, treatment time and temperature, matrix chemistry, and composite manufacturing conditions. Moderate treatment can reduce surface defects and improve fiber separation, whereas excessive alkalization can damage cellulose microfibrils, reduce the degree of polymerization, create surface pits, and decrease fiber strength [151,152]. Therefore, an optimal treatment window generally exists, and aggressive treatment can offset the benefits obtained from improved interfacial adhesion. Relatively mild NaOH treatments commonly provide the most favorable balance between surface cleaning and preservation of the load-bearing cellulose structure [161]. Alkali and silane treatments showed no significant effect on the impact strength [152]. Fiber pullout is one of the important impact energy dissipation mechanisms in composites reinforced with fibers. NaOH/silane treatment is used to enhance bonding at the interface between the fiber and the matrix. However, strong interfaces reduce the amount of pullout. The fibers are then more likely to break internally. As a result, frictional energy dissipation does not increase. However, compressive strength depends on the stress-transfer efficiency at the fiber–matrix interface. The surface of sisal fibers is waxy before treatment, resulting in gaps at the fiber–matrix interface, which prevent the effective transfer of compressive stress, making the composite material prone to failure and fracture due to interface failure.

Scanning electron microscopy (SEM) allows one to observe the changes that occur when chemically modifying natural fibers; it can provide insight into why fibers become more compatible with the matrix material. The as-received fiber contains various impurities, such as waxes and extractives, lignin, hemicellulose, pectins, and other non-cellulose materials. These impurities limit the ability of the fiber to be wetted and bond to the matrix. After alkali treatment, the fiber surface has some of these layers stripped away, resulting in a fibrous and rougher surface. Silane treatment will also cause similar changes on the surface [163,164]. It is important to look at more than just the morphology when looking at surface modifications. The changes should also be correlated to their mechanical properties, thermal properties, and resistance to water.

Surface modification using mild alkalis can increase the surface properties of natural fibers. Depending on the type of fiber used, different effects will be observable. When treating flax fibers with sodium hydroxide, it was found that the surface binder was removed along with other impurities and created a rougher fiber surface. This produced the greatest tensile modulus and strength, showing that the fiber-matrix interface had been improved [165]. When testing for water uptake, the untreated samples absorbed 3.02% of water, while the samples after being treated with sodium hydroxide absorbed 4.15% due to the increase in roughness and pores in the fiber [165]. This shows that improving adhesion may not always improve water resistance. Palmyra fiber was shown to have its extractive, hemicellulose, and lignin contents reduced when alkali-treated. Surface roughness and crystallinity increased, which decreased water absorption by 14% using 0.5 M NaOH for 60 min gave the best results [166]. Similar results were found when coir fiber was alkali-treated: impurities were removed from the fiber and increased the surface roughness. Using 5 wt.% oxalic acid for 1 h improved the tensile strength by 22.9% and showed little deterioration of the thermal properties [167].

**Figure 7:**
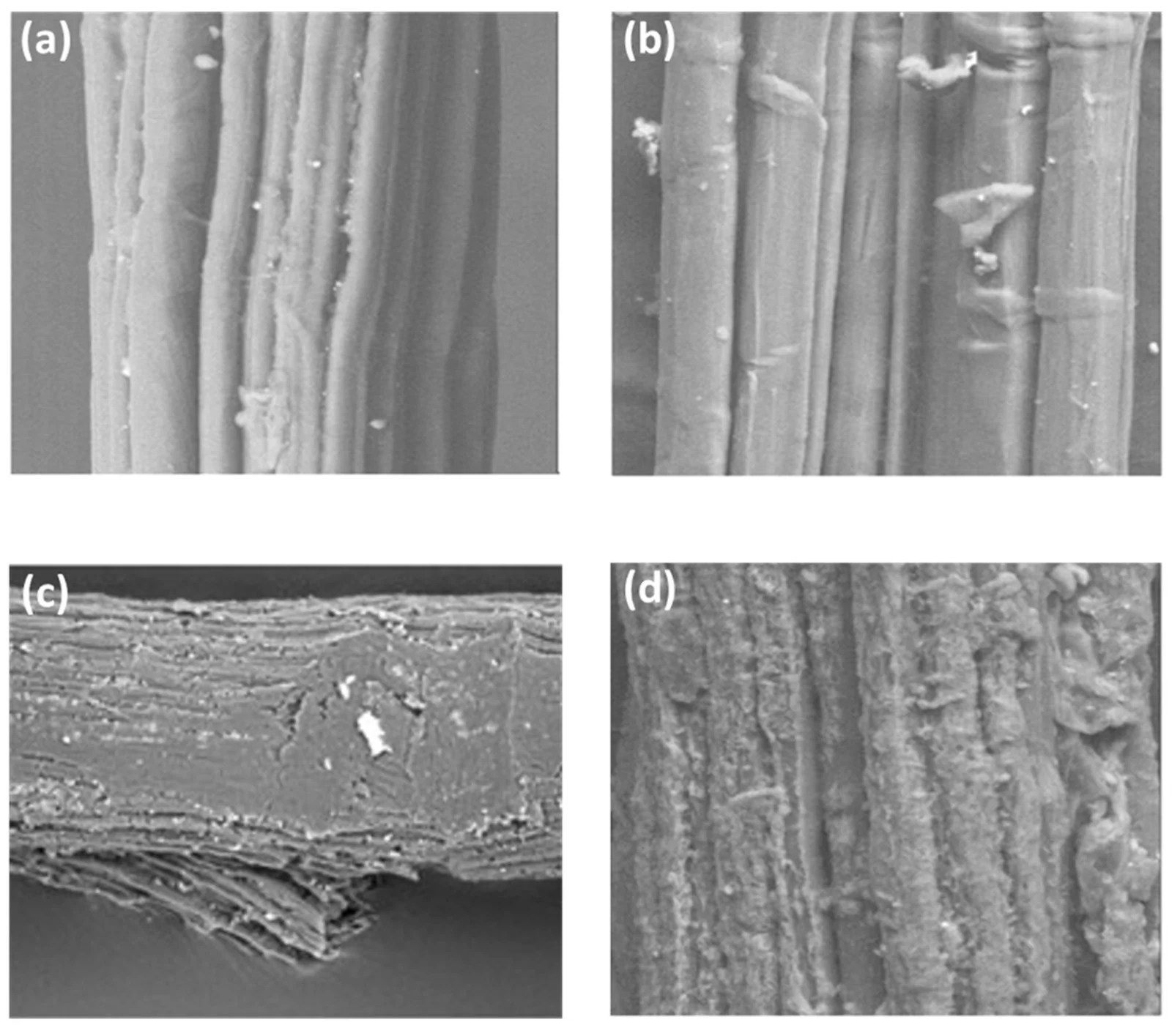
Representative SEM observations of surface morphological changes induced by chemical treatments of natural fibers: (**a**) Untreated Cryptostegia grandiflora fiber (CGF), showing surface impurities and non-cellulosic deposits; (**b**) 5% NaOH-treated CGF, showing treatment-induced roughening; (**c**) Borassus flabellifer fruit fiber treated with 15% NaOH, showing structural degradation and local fiber damage associated with excessive alkali treatment; (**d**) silane-treated CGF, showing a cleaner and strongly modified surface morphology. Panels (**a**,**b**,**d**) were adapted from Udhayakumar et al. [164] under CC BY 4.0; panel (**c**) was adapted from Boimau et al. [163] under the Creative Commons Attribution License.

Silane modification combines surface cleaning and chemical coupling. Alkylsilane-treated date. Palm fibers showed altered pore morphology and amorphous fraction removal; crystallinity rose from 31% to 41%, water contact angle from 66.8° to 116°, and thermal stability improved [168]; representative SEM images are shown in Figure 7. For Cryptostegia grandiflora fibers (CGFs), 5% NaOH increased roughness, while also increasing tensile strength from 791 to 853 MPa and crystallinity from 62.00% to 66.34%; silane-treated CGFs reached 994 MPa, 69.95% crystallinity, a lower moisture content of 8.3%, and better high-temperature resistance [164]. Thus, gains arise not from roughness alone but from the removal of weak surface layers, crystallinity changes, reduced hydrophilicity, and chemical interactions; treatment intensity must be controlled. SEM of Borassus flabellifer fruit fibers showed progressive cleaning at 5–10% NaOH, while 15% NaOH caused visible degradation and brittleness [163]. Single-fiber tensile strength rose from 430 MPa to 635 MPa at 10% NaOH, and then fell to 560 MPa at 15% NaOH [163]. Therefore, mild cleaning and roughening aid wetting and load transfer, but excessive etching damages cellulose-rich load-bearing structures and negates interfacial benefits.

SEM provides morphological evidence for interface evaluation by directly visualizing changes in fiber surface morphology. However, SEM morphology alone cannot determine interfacial quality, and it must be combined with chemical composition, crystallinity, wettability/hygroscopicity, thermal behavior, and composite mechanical/impact responses. The effect of interfacial morphology on performance is not governed by a simple rule that rougher is better. Moderate roughening and impurity removal can improve interfacial contact and mechanical interlocking and facilitate load transfer, whereas excessive etching weakens the fiber wall and increased pore accessibility accelerates moisture ingress, while coupling agents can improve moisture resistance through chemical modification without severe roughening. For energy absorbing NFRCs, an overly weak interface causes early pull-out, whereas an excessively strong or damaged interface suppresses beneficial sliding and progressive damage; therefore, interfacial morphology and strength balance must be optimized based on integrated characterization.

Recent studies further show that impact performance can be improved by tailoring interfacial toughness. Introducing a ductile cellulose-acetate interphase between flax fibers and an epoxy matrix slightly reduced the transverse tensile and in-plane shear strengths, but it increased the impact perforation energy by 42% [126]. The modified interphase changed the predominantly brittle failure of flax/epoxy laminates into a more progressive response involving greater fiber-matrix debonding. Graphene-oxide modification of flax-fiber surfaces has also been shown to improve wetting, reduce porosity, delay impact-induced fiber damage, and extend the stable damage-propagation stage. However, it did not significantly increase the final perforation energy, illustrating that improved interfacial properties may alter damage initiation and residual damage tolerance without necessarily increasing the total absorbed energy [127]. The modification promoted interfacial sliding and fiber pull-out under cyclic loading and reduced the rate of fatigue life degradation by approximately 17%, while the low-velocity impact perforation energies of the modified and unmodified specimens remained similar.

By enhancing the interfacial bonding and providing crack arresting mechanisms, particulate fillers can alter the behavior of NFRCs upon impact. Some natural fillers have been found to help fill small voids and provide crack pinning, crack deflection, debonding, and friction between particles [153]. The effects of these fillers can depend strongly on concentration. Peanut-shell powder, when added to a jute/polyester composite, gave optimal results at 6 wt.% [154], while teak wood powder/polyester composites showed the highest Charpy impact strength at 20 vol%, followed by a reduction at 30 vol% [155]. Similarly, 10 wt.% cashew-nut-shell biochar increased the impact strength of hemp/vinyl-ester composites to 37.02 J/m, approximately 38% above that of the unfilled material, whereas 15 wt.% reduced it to 28.28 J/m [156]. Palm-kernel-shell/sisal/epoxy composites also exhibited maximal impact energy and impact strength of 83 J and 47.43 kJ/m^2^, respectively, at an intermediate filler content [157].

Microscale and nanoscale fillers provide similar crack control effects. The addition of 1 vol% TiO_2_ nanoparticles increased the impact strength of jute/epoxy composites by approximately 39.35% [158]. In sisal/hemp/epoxy composites, nanosilica increased the impact energy from approximately 1.1 J without filler to 1.6 and 2.1 J at 3 and 6 wt.% SiO_2_, respectively; further addition reduced the impact performance by approximately 23.5% [159]. Nevertheless, particulate addition does not invariably enhance energy absorption. In sisal fiber/groundnut-shell-powder/epoxy composites, the fiber only system exhibited an impact energy of 0.4 J, whereas particle containing composites generally achieved only 0.2–0.3 J [158], indicating that replacing fibrous reinforcement with rigid particles may suppress effective fiber bridging and pull-out mechanisms.

Particulate fillers generally exhibit a non-monotonic effect on energy absorption. At low-to-moderate concentrations, well dispersed particles can improve interfacial integrity, increase crack-path tortuosity, and dissipate energy through crack deflection, particle debonding, and matrix deformation. However, excessive filler loading promotes particle agglomeration, increased resin viscosity, poor fiber wetting, void formation, and matrix embrittlement, ultimately reducing the impact resistance. Therefore, optimizing particle concentration, morphology, dispersion, and interfacial compatibility is more important than simply increasing the filler content when designing NFRCs for energy-absorbing applications.

### 4.2. Reinforcement Architecture and Laminate Design

#### 4.2.1. Reinforcement Form and Textile Architecture

Reinforcement form and textile architecture strongly influence load transfer, damage propagation, and energy dissipation in NFRCs. Common configurations include unidirectional (UD), non-woven, 2D woven, and 3D woven or stitched architectures. Compared with fiber type, textile architecture mainly controls fiber alignment, yarn crimp, interlacement, and through-thickness constraint, thereby governing whether impact damage develops through localized fiber fracture or more distributed mechanisms such as yarn straightening, sliding, crack deflection, and delamination [169,170].

**Figure 8:**
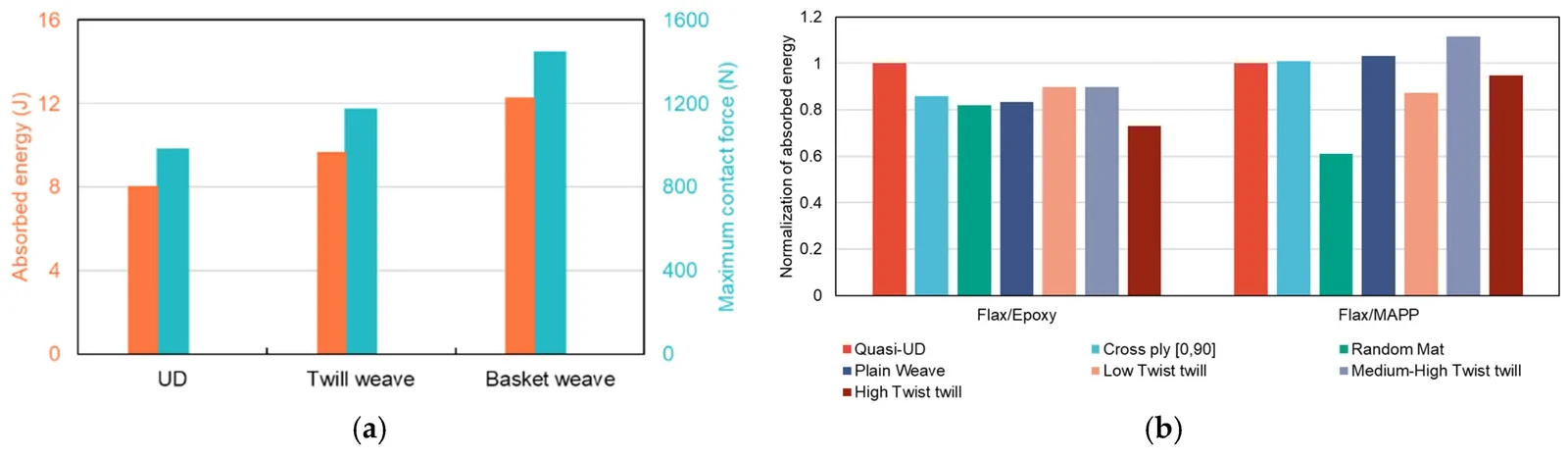
(**a**) Flax/PLA composites with 3 different architectures under 15 J in low-velocity impact (data were sourced from Ref. [171]). (**b**) Normalization of absorbed energy for flax/epoxy and flax/MAPP composites with different architectures in low-velocity impact (data were sourced from Ref. [129]).

UD architecture provides highly efficient load transfer because of their low fiber crimp and high alignment, resulting in high longitudinal stiffness and strength. However, their limited transverse constraint can promote localized damage. UD, basketweave, and twill-weave flax/PLA composites were compared as shown in Figure 8a. Although UD laminate exhibited the highest tensile strength and modulus in the longitudinal direction, the basketweave laminate showed the greatest contact force and impact energy absorption [171]. The UD specimens also developed larger impact-damaged areas, indicating that high fiber alignment does not necessarily provide the best impact tolerance. Normalization of absorbed energy for flax/epoxy and flax/MAPP composites with different architectures in low-velocity impact is shown in Figure 8b. It can be seen that the influence of architecture on energy absorption characteristics is also coupled with matrix type. Among the various architectures, quasi-UD flax/epoxy composites exhibit the highest energy-absorption capability, whereas flax/MAPP composites do not show the same trend.

Woven architectures offer better multidirectional load redistribution through warp-weft interlacement. During impact, yarn straightening, rotation, inter-yarn friction, and pull-out provide additional energy-dissipation mechanisms. Nevertheless, excessive crimp reduces the effective stiffness of the fibers and creates concentrations of local stress. A 3/1 twill jute/polyester architecture achieved substantially higher impact strength than plain and other twill configurations, indicating that an appropriate balance between yarn float length and interlacement density is important [172]. Similar comparisons of woven, woven-commingled, and knitted-commingled natural-fiber composites also demonstrated that more deformable architectures could improve impact resistance or compression-after-impact performance by promoting progressive deformation and improving impregnation [173,174].

Through thickness reinforcement further alters impact behavior by suppressing unstable delamination. Stitching woven kenaf/epoxy laminates increased impact performance by ~33% compared with unstitched laminates [175]. Three-dimensional woven jute/green epoxy composites showed that binder-yarn trajectory strongly affects absorbed energy and residual properties: orthogonal through-thickness structures provided high impact strength, whereas angle interlock and hybrid architectures promoted higher absorbed energy or compression after impact performance [176]. More recent work similarly showed that 3D architectures with longer yarn floats and lower crimp can outperform highly crimped interlock structures under several impact-loading modes [177].

Overall, reinforcement architecture governs energy absorption through a trade-off between fiber alignment and architectural mobility. UD reinforcement maximizes initial load-bearing efficiency, whereas woven, knitted, stitched, and 3D architectures activate additional mechanisms such as yarn straightening, inter-yarn friction, crack deflection, and controlled delamination. Therefore, effective energy-absorbing architectures should combine sufficient fiber alignment for load-carrying with adequate in-plane interlocking and through-thickness constraint to promote stable and progressive damage.

#### 4.2.2. Stacking Sequence

It has been shown that the stacking sequence plays a major role in the effects and energy absorption of natural fiber composites. It can affect how the laminate bends, the amount of stress that the plies experience, and the path in which cracks may form. Having surface plies running longitudinally can provide a stiffer composite. However, this can cause large amounts of stress to be put onto the opposite side of the impacted face, which may lead to matrix cracking and fiber breakage. The use of a ply with fibers running transversally on the surface would allow more deflection and distribute stress to the other plies, allowing for less local failure [178].

It is also significant what angles are used in between plies. If a cross-ply configuration was used, it could help stop crack growth because the ply next to it would have an angle of 90 degrees, allowing for more strength in different directions. When using cross-ply flax/epoxy laminates, multiple low-velocity impacts showed that the damage pattern consisted of back-face cracking and then delamination as the impact energies and number of impacts increased [179]. Using more multidirectional stacking sequences can increase the damage path by deflecting the cracks and having different cracks interact with one another; this has been found to increase the amount of energy dissipated through delamination [35].

Recent experiments further demonstrate that no single stacking configuration simultaneously maximizes all impact performance indicators. Cross-ply, angle-ply, and quasi-isotropic flax/epoxy laminates were compared under drop-weight impact [180]. The angle-ply configuration exhibited impact resistance approximately 3.5% and 6.45% higher than that of the cross-ply and quasi-isotropic configurations, respectively; these differences increased to approximately 9.31% and 19.11% at an impact energy of 15 J. However, post-impact flexural strength decreased by approximately 14% for the cross-ply laminate and 26% for both the quasi-isotropic and angle-ply configurations [180]. These results indicate that a layup providing high resistance during impact does not necessarily retain the highest residual mechanical performance.

The number of plies and laminate thickness provide another important design parameter. Increasing the ply number generally increases bending stiffness and peak impact force while reducing maximum displacement [59]. In tubular crash absorbers, additional layers also increase the total energy absorption because a larger amount of material participates in progressive deformation and fracture; however, this benefit is not proportional to thickness. Excessive wall thickness can cause the initial peak crushing force to increase more rapidly than the mean crushing force, reducing crushing-force efficiency and increasing the peak load transmitted to the protected structure [9]. For jute composite tubes, for example, the total energy absorption increases with the number of layers, whereas specific energy absorption may initially increase and subsequently decrease because the gain in absorbed energy is eventually offset by the increase in structural mass [43]. This behavior suggests the existence of an optimal ply number.

Hybrid fiber-reinforced composites (HFRCs) can not only resist the initial impact force but also absorb the remaining energy by combining fibers with high rigidity and good toughness through plastic deformation, which can avoid the limitations of a single kind of fiber [151,153]. A composite material composed of 55% ramie and 45% jute was found to have the optimal synergistic effect, enhancing the overall energy-absorption efficiency [181]. In the layup design, the hybrid composite material uses high-modulus fibers as the skin layer to resist the initial impact force, while using fibers with good toughness as the core layer to absorb the remaining energy through plastic deformation. From the perspective of mechanical mechanism, ramie fibers have a dense structure, leading to high tensile strength and modulus. The multi-cavity structure of jute fibers can absorb more energy through fiber fracture. Organizing the synthetic-fiber layer on the outer layer and the natural-fiber layer on the inner layer is recommended when mixing layups with synthetic fibers [182,183], because synthetic fibers possess stronger impact resistance and the inner natural-fiber layers have good toughness, which can assist in absorbing energy through their own deformation during impact and reduce the direct force loss of the outer layers [184]. The synergistic effect of the two makes the impact energy-absorption capacity of this type of composite material optimal. Although natural fibers can only withstand tensile stress, when arranged below the neutral axis, they can effectively bear the tensile force under bending loads, reduce tensile damage to the matrix, and delay the failure process of composite materials [185].

Overall, stacking sequence design involves a trade-off among stiffness, deformation capacity, crack-path control, delamination, peak load, and structural mass. Cross-ply laminates provide effective multidirectional load transfer, whereas angle-ply and quasi-isotropic configurations can promote crack deflection and distributed damage. Meanwhile, excessive ply number may increase total absorbed energy at the expense of *SEA* and crushing-force efficiency. 

#### 4.2.3. Fiber Orientation

The fiber orientation has a large effect on the impact response and energy absorption of natural-fiber composites, because fiber orientation can affect how load is carried through the material, the amount of anisotropy within the laminate, the direction of crack propagation, and what failure modes occur, such as fiber fracture, matrix cracking, debonding, and fiber pull-out. When fibers are oriented in the direction of loading, they will exhibit high stiffness and strength due to the load being transferred through the length of the fiber. As the angle of the fiber increases from this orientation, the load-carrying capacity of the fiber decreases. The matrix will now carry more shear, and there will be more deformation at the interface. Fiber rotation and crack deflection will also come into play. Therefore, the orientation of the fiber that provides the best impact load may not give the greatest energy absorption [186,187].

**Figure 9:**
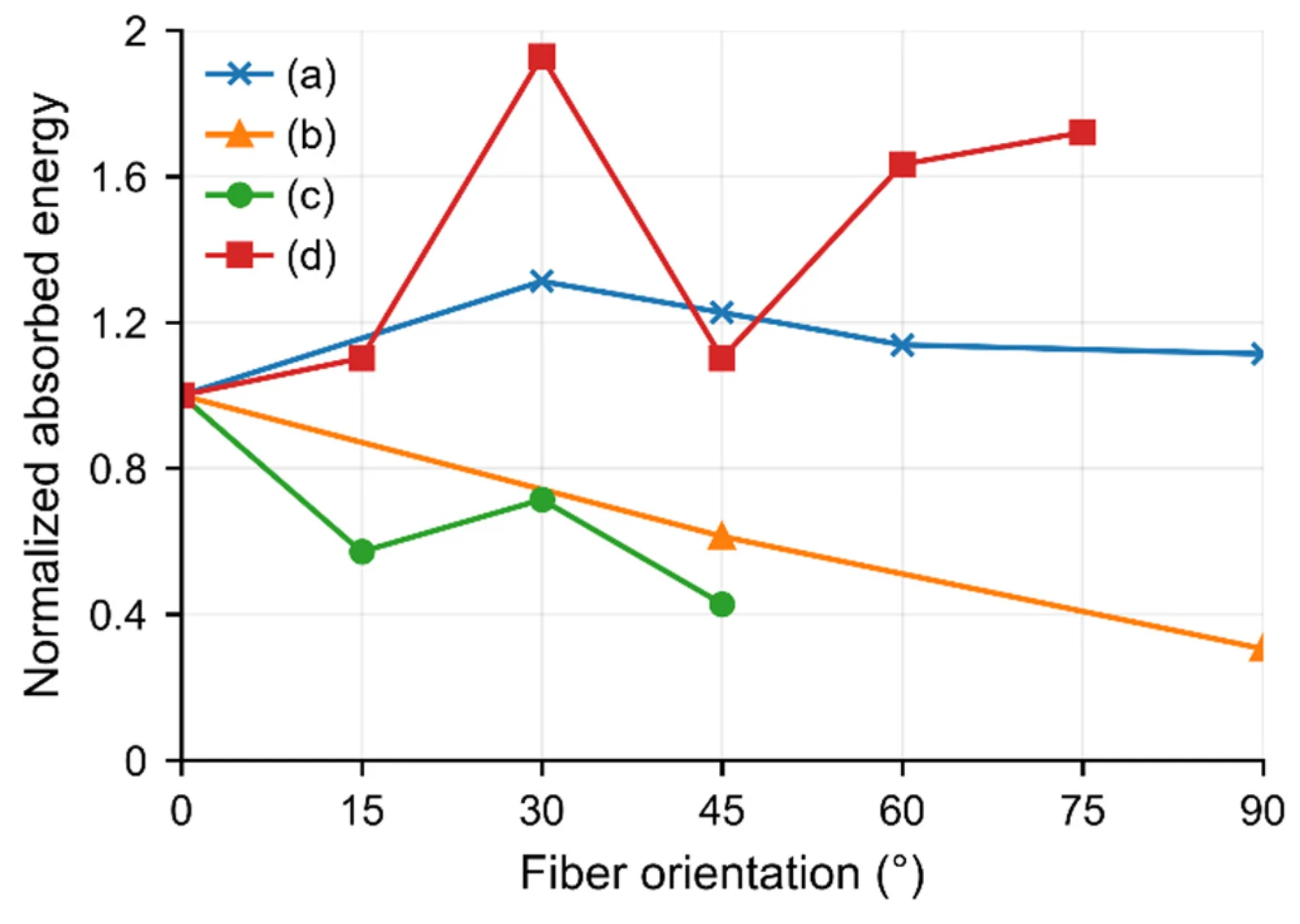
Normalization of absorbed energy for (a) Flax/PP composites under drop-weight impact test, (b) bamboo/polyester composites under pendulum impact test, (c) kenaf/epoxy composites under pendulum impact test, and (d) woven jute/epoxy composites under pendulum impact test (data were sourced from Refs. [186,187,188,189]).

This loading mode dependence was clearly demonstrated for UD flax/polypropylene composites, as shown in Figure 9 [186]. The 30° specimens absorbed the greatest amount of energy, with measured absorbed energies ranging approximately from 20 to 32 J under drop-weight impact. The off-axis fibers provided additional deformation and redistributed the impact-induced stresses, thereby changing the crack trajectory and failure mode. In contrast, the 0° specimens exhibited the highest energy absorption because fibers aligned with the loading direction effectively resisted crack opening and carried the impact load under Charpy impact [186]. The other composites displayed in Figure 9 generally exhibited higher impact energy at smaller fiber orientation, whereas woven jute/epoxy reached its maximum at an intermediate orientation of approximately 30° [187,188,189]. The optimal fiber orientation for energy absorption is not universal but depends on the loading mode and reinforcement architecture.

Multidirectional fiber arrangements can further improve damage tolerance by interrupting preferential crack paths. In UD laminates, cracks tend to propagate along fiber–matrix interfaces or weak transverse directions, resulting in strongly localized damage. Introducing fibers in a second direction can impede this continuous propagation and activate crack deflection, fiber bridging, and interfacial friction. Accordingly, cross-ply natural fiber laminates can exhibit greater resistance to crack growth than corresponding UD laminates [144]. A recent low-velocity impact study similarly identified fiber orientation as a major parameter controlling peak force, damage development, delamination, and energy absorption in NFRCs [69]. Under repeated impact, cross-ply flax/epoxy laminates have also been observed to develop progressive cracking followed by increasing delamination [37,184].

Overall, optimization of fiber orientation represents a trade-off between load-bearing efficiency and damage-mediated energy dissipation. Orientations close to the principal loading direction favor stiffness and peak load resistance, whereas moderate off-axis orientations can promote shear deformation, crack deflection, interfacial sliding, and progressive damage. Multidirectional arrangements can further suppress preferential crack propagation and stabilize the overall response. Therefore, the optimal orientation should be selected according to the loading direction and design objective.

#### 4.2.4. Fiber Content

Fiber content is an important parameter governing the mechanical response and energy-absorption behavior of NFRCs. Within an appropriate range, increasing the fiber content can improve damage resistance and mechanical properties [10,190]. A high fiber volume fraction facilitates the formation of multiple, diverse energy dissipation pathways, mitigates premature cracking in resin rich regions, promotes stable and progressive energy absorption, and consequently leads to a substantial improvement in specific energy absorption [150]. For example, increases of approximately 13% and 58% in specific energy absorption were reported for cotton/epoxy and jute/epoxy composites, respectively, with increasing fiber content [185]. Short alfa-fiber composites exhibited maximum energy absorption and a relatively small damage area at a fiber content of approximately 50% [191]. Nevertheless, the influence of fiber content is strongly dependent on the loading mode. In flax/PP composites, increasing the fiber content enhanced in-plane impact-energy absorption but reduced out-of-plane absorbed energy [186].

The optimal fiber content also varies considerably among different fiber/matrix systems. Maximal energy absorption was reported at approximately 5% bagasse fiber and 10% coconut-shell and sisal reinforcement [192]. Similarly, for epoxy composites reinforced with 10–30 vol.% Geonoma baculifera fibers, the impact energy increased from 21.86 J/m^2^ at 10 vol.% to 88.34 J/m^2^ at 30 vol.% [193]. Microscopic observations showed increasingly evident fiber pull-out, interfacial debonding, fiber rupture, and crack deflection with increasing fiber content. This suggesting that the improvement in impact resistance resulted from the activation of different fracture and dissipation mechanisms in addition to fiber strength alone.

**Figure 10:**
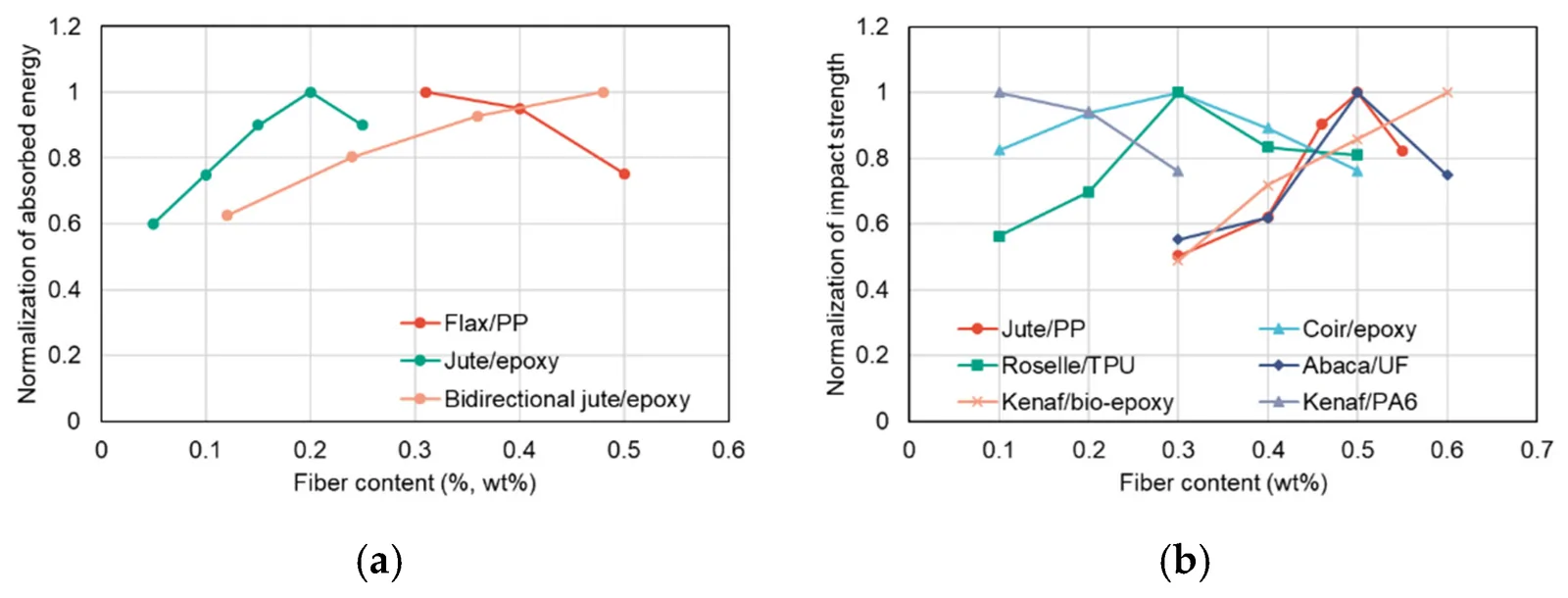
Effect of fiber content on the energy-absorption behavior of NFRCs: (**a**) normalized absorbed energy of flax/PP, jute/epoxy, and bidirectional jute/epoxy composites [186,194,195]; (**b**) normalized impact strength of different NFRCs as a function of fiber content [196,197,198,199,200,201].

Figure 10 further illustrates how energy absorption behavior varies with fiber content. As shown in Figure 10a, the absorbed energy of flax/PP decreased with increasing fiber volume fraction, whereas jute/epoxy exhibited either an optimum at approximately 20 wt.% or a continuous increase in the case of bidirectional reinforcement [186,194,195]. Likewise, Figure 10b shows that impact strength generally exhibits a nonlinear dependence on fiber content [196,197,198,199,200,201]: several systems display an intermediate optimum, while kenaf/bio-epoxy increases continuously and kenaf/PA6 decreases with increasing fiber loading. These results demonstrate that the effect of fiber content on energy absorption is not universal but arises from a competition between the increasing number of fiber-related dissipation mechanisms and changes in matrix fraction, fiber wetting, interfacial load transfer, and defect formation.

These observations suggest an optimal fiber/matrix ratio at which sufficient reinforcement is available to dissipate energy. At the same time, enough matrix remains for fiber wetting, stress transfer, and plastic deformation. Beyond this optimum, fiber crowding and agglomeration, insufficient resin impregnation, higher void content, and reduced inter-fiber spacing can promote premature debonding and delamination. This reduces the overall energy absorption efficiency. Accordingly, the relationships among fiber content, impact fracture behavior, and absorbed energy are generally nonlinear [39,142,202].

### 4.3. Environmental Conditions and Long-Term Durability

Due to the hydrophilic nature of natural fibers, one concern is their behavior under moist environments. Water absorption may cause significant changes to the stiffness, strength, damage progression, and energy-absorption capabilities [203,204,205], usually behaving as a Fickian diffusion process where the moisture content at equilibrium will vary with fiber content and the relative humidity of the environment [116,206]. During normal usage, moisture will not be the only environmental condition: heat will cause moisture migration and degradation of the matrix and interface, UV exposure may lead to oxidation of surfaces and cracking, and impact loading can cause progressive damage to the matrix cracking, interface debonding, and delamination. Long term durability should be considered as the evolution of impact and energy absorption performance under coupled environmental and mechanical exposure. Moisture exposure itself is also non-monotonic. Moderate uptake may increase apparent toughness through plasticization, whereas prolonged exposure can cause irreversible interfacial and structural degradation [207,208].

Plant fibers contain abundant hydroxyl groups and, therefore, exhibit strong hydrophilicity [116,209]. Water absorption causes fiber swelling and weakens fiber-matrix interfacial bonding, which can promote interfacial debonding and reduce load-transfer efficiency [210,211]. In addition, the leaching of water soluble constituents from natural fibers may further deteriorate interfacial integrity [211,212]. Nevertheless, moderate moisture uptake may increase deformation capacity through plasticization. Water increases molecular mobility and reduces brittleness in both natural fibers and susceptible polymer matrices, thereby increasing failure strain and fracture energy even when stiffness and strength decrease [116,207,208]. Increased interfacial sliding, fiber pull-out, crack deflection, and matrix deformation can therefore provide additional energy-dissipation mechanisms during impact [212,213].

**Figure 11:**
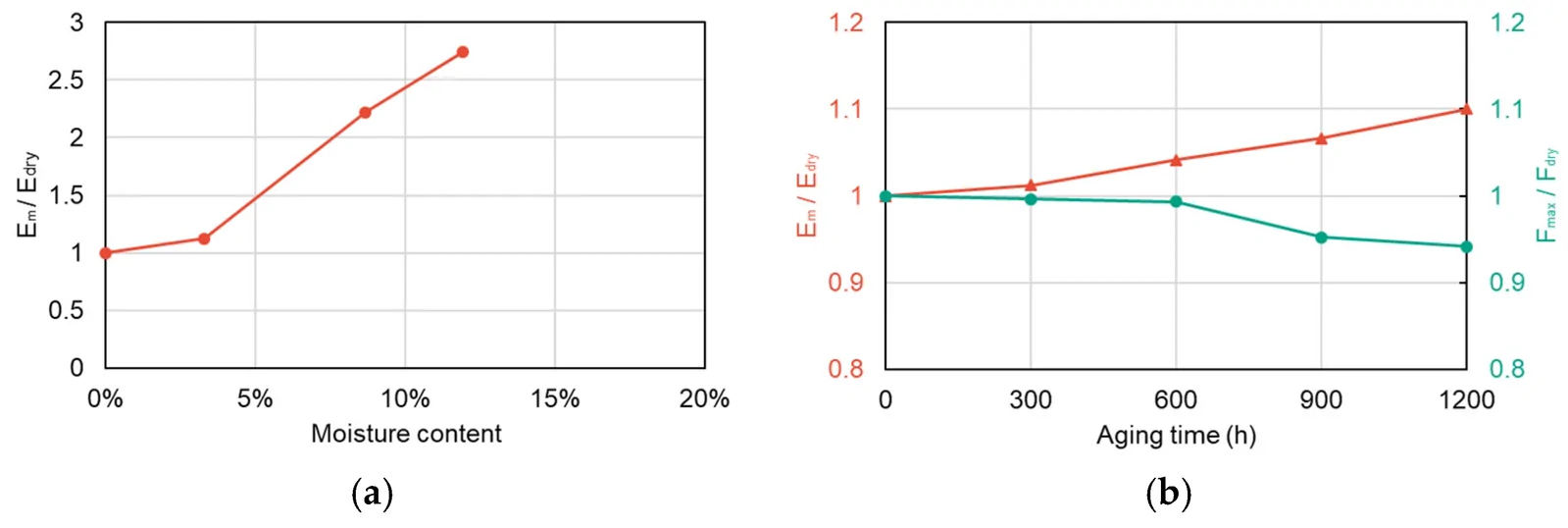
Effects of moisture and aging on the impact energy absorption of NFRCs: (**a**) normalized energy absorption response of flax/PLA composites at different moisture contents [207]; (**b**) normalized absorbed energy and tolerated impact load of flax/epoxy laminates before and after 1200 h of combined UV–humid aging [214].

The competing effects of moisture on impact response are illustrated in Figure 11. Flax/PLA composites at moisture contents of approximately 0, 3.28, 8.66, and 11.9% were investigated. The energy-absorption capability increased markedly with moisture content. The absorbed energy limit increased from approximately 13.5 J in dry conditions to approximately 37 J at 11.9% moisture content. This behavior demonstrates a characteristic strength–toughness trade-off. Moisture-induced plasticization reduces rigidity and load-bearing strength but permits larger deformation before failure, thereby increasing the energy dissipated through matrix deformation, interfacial sliding, fiber pull-out, and crack deflection. Consequently, an increase in absorbed energy under moderate moisture exposure should not necessarily be interpreted as an improvement in structural impact resistance. This distinction is further demonstrated in Figure 11b for flax/epoxy laminates subjected to combined UV and humid aging for 1200 h [214]. After aging, the impact load decreased by approximately 6%, whereas the measured absorbed energy increased by approximately 10%. The reduction in load bearing capacity and increase in absorbed energy indicate that the additional energy was dissipated mainly through more extensive damage. Interfacial degradation caused by moisture can enlarge the damaged region and promote delamination, interfacial sliding, and fiber pull-out, thereby increasing the measured energy absorption despite a deterioration in impact resistance.

**Figure 12:**
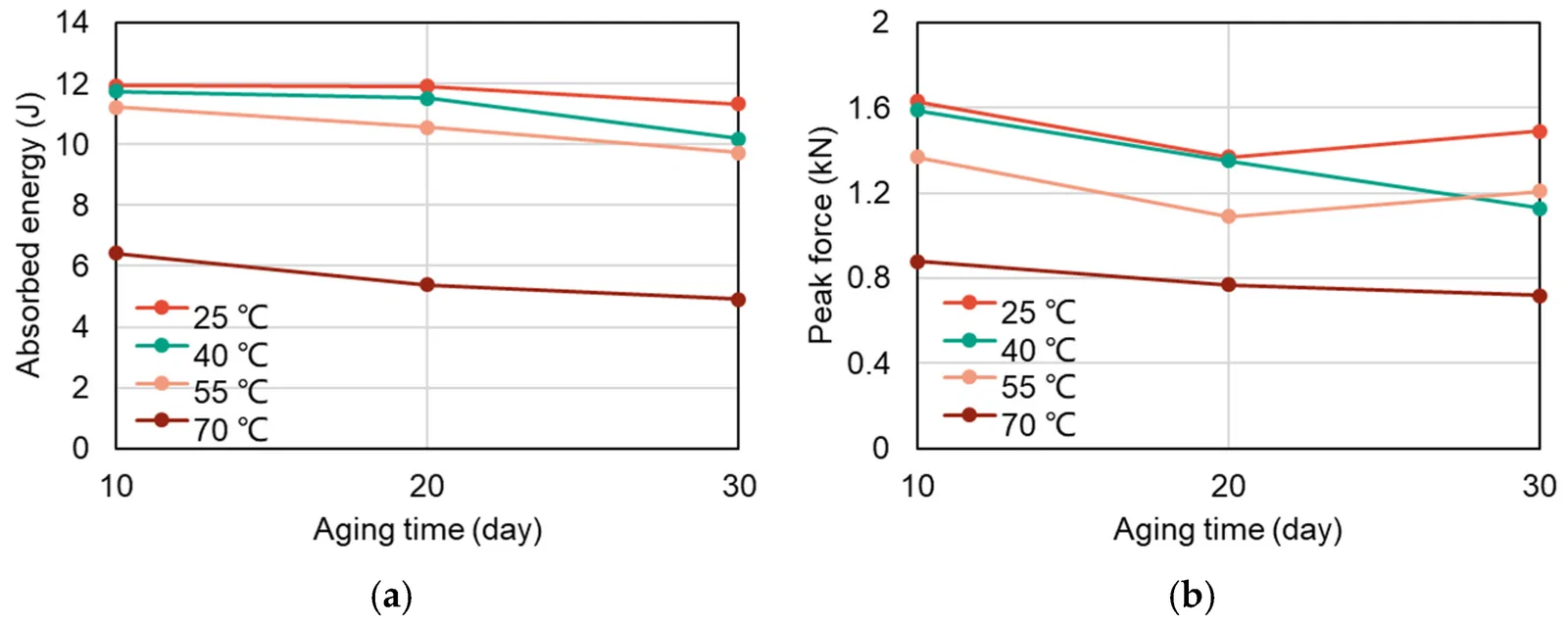
(**a**) Absorbed energy and (**b**) peak force of flax-fiber-reinforced foam sandwich panels after water/hygrothermal aging at different temperatures and exposure durations [215].

When the severity of exposure is increased, it is possible for irreversible effects to take over from the plasticization effect. As illustrated in Figure 12, flax-fiber-reinforced foam sandwich panels subjected to water and hygrothermal aging exhibited a progressive deterioration in impact energy absorption with increasing aging severity [215]. After being aged for 30 days at 70 °C, there was a 56.7% decrease in energy absorbed when compared to a specimen that was aged at 25 °C when impacted at 12 J. Higher temperatures allow for moisture to diffuse through the material faster, causing fibers to swell and degrade the matrix. This can lead to loss of adhesion, microcracking, and delaminations, all of which will cause the structure to have less ability to absorb impact energy through progressive damage. Therefore, moisture’s effect on the energy absorption of NFRCs can be seen as a nonlinear process that is dependent on the severity of exposure. Moisture may cause the structure to appear tougher due to plasticization and increased deformation, allowing it to absorb more energy. However, when exposed to higher temperatures and moisture levels for longer periods of time, the material can experience degradation of the interface, matrix degradation, fiber degradation, and the formation of voids and delaminations [207,214,216]. The balance between these competing mechanisms determines whether the measured absorbed energy increases or decreases.

Repeated impact introduces an additional durability concern because damage generated during an initial event modifies the stiffness, stress redistribution, and failure pathways during subsequent loading. Repeated low-velocity impact tests on cross-ply flax/epoxy laminates showed progressive accumulation of cracking and delamination with increasing impact number and energy [179]. Matrix selection also plays an important role. Flax composites based on bio-based epoxy and recyclable thermoplastic matrices exhibited greater repeated impact damage resistance than conventional epoxy systems in the investigated configurations [217]. These effects become particularly relevant after environmental aging because moisture-, temperature-, and UV-induced degradation can weaken the matrix and fiber-matrix interface, thereby modifying the subsequent impact-damage evolution [218]. However, systematic studies combining repeated impact with multi-factor environmental aging remain limited [218]. Long-term durability assessment should therefore move beyond single factor conditioning toward coupled impact protocols, with performance evaluated using peak force, EA/SEA retention, residual strength, damage area, and impacts to failure.

Coir/polypropylene illustrates that alkaline-silane treatment reduced water absorption and thickness swelling and improved tensile and flexural properties compared with untreated or alkali-only systems [219]. The benefits stem from fewer hydrophilic groups and better interfacial compatibility, although concentration and processing must be controlled. Silane-treated palmyra-leaf-stalk/polyester composites had lower moisture uptake and better tensile, flexural, and impact performance than untreated or alkali-treated systems after water exposure [220]. Alkali treatment removed extractives, hemicellulose, and some lignin, increased crystallinity, reduced water absorption, and improved mechanical properties, with intermediate durations proving best [166]. Wool differs because keratin has hydrophilic and hydrophobic regions. Moisture sorption causes dimensional, morphological, and mechanical changes affecting manufacture and service [9]. Plasma or acetylation can adjust hydrophilicity, but strong alkali treatments are unsuitable for wool due to protein degradation risk [139,221].

Several strategies have been proposed to improve the environmental durability of NFRCs. Hybridization with synthetic fibers can reduce moisture uptake and retard moisture-induced mechanical degradation [205,222,223], whereas surface modification, compatibilizers, nanofillers, and protective coatings can restrict water ingress and preserve fiber–matrix interfacial integrity [224,225,226,227]. Bio-based carbon fillers have also been reported to reduce water absorption by approximately 40% while improving flexural strength and fracture energy [228]. However, the effectiveness of these strategies should be validated under coupled aging conditions. The long term durability of NFRCs is governed by interactions among moisture diffusion, temperature, UV-induced degradation, interfacial evolution, and accumulated mechanical damage.

### 4.4. Structural Geometry and Crashworthiness Design

The geometry of the structure has a significant impact on the crashworthiness of natural-fiber composites. It can affect how stresses are applied to the part and allow for more stable deformation patterns. When looking at standard shapes that are commonly used, circular cross-sections will usually give a more stable axial crushing response then square or rectangular ones, due to there being no corners, which create large amounts of stress around the perimeter of the tube, potentially resulting in higher SEA and CFE values, as seen in some applications [16,37]. Square tubes have also been found to be beneficial due to their simplicity. Previous research has found that decreasing the angle of a cone shaped tube can achieve similar results to a cylindrical tube, providing a more stable axial crushing response and increasing the energy-absorption values [37]. The angle of a cone should not be looked at as an independent variable, as there are other variables that come into play.

Another variable to look at when designing the structure is the diameter and thickness of the tube. Having a thicker tube can increase the overall stiffness of the structure and reduce wall buckling, allowing for more of the tube to crumple during impact. It has been found that decreasing the diameter-to-thickness ratio (D/t) can lead to an increase in SEA [37]. Studies have shown that increasing the thickness-to-diameter ratio past 0.04 can help achieve a progressive crushing response instead of a buckle failure [8]. Excessive wall thickness may also increase the initial peak crushing force and structural mass, thereby reducing CFE or SEA despite increasing the total absorbed energy. Geometric triggers can further stabilize the crushing process by deliberately defining the location of damage initiation. Recent experiments on jute/epoxy circular tubes demonstrated that strategically introduced holes can significantly alter the maximal crushing stress, EA, and SEA, with tube length, hole diameter, number, and position interacting to determine the final crashworthiness response [229]. Similarly, flax/epoxy and flax/carbon hybrid tubes showed that trigger configuration strongly influences crushing initiation and the subsequent energy-absorption process [36].

The primary role of structural design is not simply to increase structural stiffness or total absorbed energy but to regulate damage initiation and maintain a stable progressive crushing mode with a high mean crushing force and a controlled initial peak load. Brittle splitting and sudden fracture produce sharp load drops and inefficient utilization of the available crushing stroke [43], whereas controlled fragmentation, splaying, folding, and progressive delamination maintain a more stable mean crushing force.

## 5. Sustainability–Performance Assessment

### 5.1. NFRCs and the United Nations Sustainable Development Goals

As mentioned above, NFRCs can play a role in sustainability not only by decreasing the global warming potential on the material scale but also by aligning with multiple UN SDGs. NFRCs have been associated with many UN SDGs, but mainly SDG 9 and SDG 12, as they allow for greater usage of resources and decrease synthetic reinforcement usage while also allowing for cleaner production methods [1]. Although these are goals that NFRCs can aim to achieve, success is not guaranteed. Whether they are truly sustainable depends on how the fibers are grown or obtained and processed, as well as the matrix with which they are paired. Factors such as the energy used during production, the lifespan of the material, and disposal methods will also determine whether NFRCs contribute to any of the UN SDGs.

The strongest alignment lies with SDG 12: Responsible Consumption and Production. Target 12.2 advocates the sustainable management and efficient utilization of natural resources, while Target 12.5 seeks waste reduction via prevention, minimization, recycling, and reuse [230]. NFRCs enable the replacement of certain energy intensive synthetic fibers with renewable lignocellulosic feedstocks, converting agricultural and industrial residues into value added materials. Byproducts including coir, rice husks, wheat straw, banana residues, and nutshells can be processed into reinforcing fibers, fillers, or biopolymer precursors [231]. Such approaches are well aligned with Target 12.5 when genuine waste streams are adopted. Nevertheless, these environmental benefits diminish if fiber extraction requires energy-intensive drying, purification, or chemical treatments or generates additional waste.

NFRCs are also linked to SDG 9: Industry, Innovation and Infrastructure, particularly Target 9.4 on improved resource efficiency and broader adoption of clean, eco-friendly industrial technologies [232]. In optimized designs, replacing glass/carbon fibers with low-impact natural fibers lowers the environmental and energy footprint of composite manufacturing. For instance, kenaf-fiber composites replacing automotive GFRCs maintain comparable specific mechanical properties, reducing production energy by 33.1% and total environmental impacts by 22.8% [233]. However, this outcome is not universal for all NFRCs; processing routes, power sources, resin selection, surface modification, production waste, and manufacturing scale alter environmental performance. Evaluations must account for raw materials, fabrication, service, and end-of-life phases [234].

The link to SDG 7: Affordable and Clean Energy is more conditional, especially Target 7.3, which aims to double the global rate of energy-efficiency improvement by 2030 [235]. NFRCs may help indirectly by lowering production energy and enabling lightweighting. Lower-density parts can reduce transport mass and may lower use-phase energy demand, and some natural-fiber systems need less non-renewable production energy than conventional synthetic-fiber composites. A life-cycle assessment of coir-fiber automotive composites reported lower environmental impacts than comparable glass-fiber composites, but energy and greenhouse-gas savings from lightweighting depend on vehicle technology and category [236]. Therefore, the contribution to Target 7.3 is application-dependent and should be shown at the component or system level, not inferred from low fiber density.

NFRCs may also support SDG 13: Climate Action if replacing conventional materials gives demonstrable life-cycle greenhouse-gas reductions. However, this link is less direct than those with SDG 9 and 12. SDG 13’s targets mainly concern climate resilience, policy integration, planning, finance, and institutional capacity. Target 13.2, for example, addresses integrating climate-change measures into national policies, strategies, and planning [237]. A material technology cannot meet such a policy target alone; NFRCs should therefore be seen as one possible contributor to industrial decarbonization, not as a direct route to SDG 13. Their climate benefit needs consistent life-cycle boundaries because polymer matrices, manufacturing, chemical treatments, durability, replacement frequency, recycling, and end-of-life scenarios can offset some advantages of natural fibers [1,234].

Overall, NFRCs contribute most to the SDGs when renewable or waste derived feedstocks, low-impact processing, adequate durability, lightweight structural performance, and circular end-of-life pathways are combined, renewable reinforcement alone does not guarantee sustainability. This matches the SEA production-related GWP assessment in this review. NFRCs can occupy a favorable performance and environment region, but the advantage depends on the full formulation and production route. Future work should move from maximizing natural-fiber content toward normalized performance life-cycle optimization, integrating mechanical efficiency, energy absorption, manufacturing impacts, durability, resource circularity, and end-of-life options [1,234,236].

### 5.2. Data Collection, Normalization, and Environmental Burden of Reinforcement and Composite Systems

For the SEA-GWP comparison, mechanical performance data were collected only when the material system, specimen configuration, loading mode, and reported or derivable SEA could be identified with sufficient clarity. All SEA values were converted to a common unit of kJ/kg. While energy absorption and specimen mass were reported separately, SEA was calculated as the absorbed energy divided by specimen mass. The corresponding loading condition was retained in the database, including quasi-static crushing, low-velocity impact, and dynamic loading where applicable. Because SEA is strongly influenced by specimen geometry, loading rate, boundary conditions, triggering features, crushing displacement, and failure mode, it was not treated as an intrinsic material property; instead, the collected values were used as representative engineering performance indicators for broad comparative screening. Data points were excluded when the material composition, specimen mass, absorbed energy, or loading condition could not be established reliably.

NFRCs are commonly associated with the renewable origin and relatively low production energy of lignocellulosic reinforcements. However, the environmental performance of an energy-absorbing composite cannot be inferred from the reinforcement alone. GWP depends on fiber and matrix production, constituent fractions, surface treatments, manufacturing routes, transportation, electricity mix, allocation procedures, and the system boundary adopted in LCA. Recent reviews have shown that differences in functional units, system boundaries, databases, and modeling assumptions can substantially affect comparisons among composite systems [238]. GWP values should be interpreted as indicators that depend on the conditions considered.

For the sustainability performance assessment in this review, cradle-to-gate GWP, expressed in kg CO_2_-eq per kg of composite, is considered the preferred environmental indicator because it is directly compatible with the mass-specific basis of SEA. Ideally, each reported SEA value should be paired with the GWP of the same composite formulation. GWP*_f_* is useful for preliminary environmental screening but represents only part of the total composite burden. GWP*_f_* is influenced by cultivation, extraction, retting, drying, transportation, and co-product allocation [239]. Likewise, synthetic fibers can exhibit considerable variability; carbon-fiber production, for example, requires energy intensive precursor preparation, stabilization, and carbonization, resulting in substantially higher GWP than most natural fibers, while the final value remains strongly dependent on process efficiency and electricity supply [30].

The matrix and manufacturing route can be equally important. Composite-level GWP values of approximately 1.19 and 1.70 kg CO_2_-eq kg^−1^ were obtained for flax/PLA and hemp/PLA, respectively, compared with 9.14 kg CO_2_-eq kg^−1^ for the investigated glass-fiber/PA66 composite [240]. Recent studies show that fibers are not necessarily the greatest source of environmental impact. For a flax-fiber composite laminate, resin production and vacuum infusion together contributed a substantial proportion of the total GWP [241]. Similarly, a recent assessment of hemp/glass/polypropylene composites found that polypropylene dominated the environmental burden, although partial replacement of glass fiber with hemp still reduced the overall GWP [242]. These studies demonstrate that a low-*GWP_f_* reinforcement does not automatically result in a proportionally low composite-level GWP.

Manufacturing can further alter the environmental rankings. Composite systems with high curing temperatures, long processing times, high scrap rates, or energy-intensive fabrication may lose part of the environmental benefit associated with renewable constituents. Recent studies comparing flax- and glass-fiber composites have also shown that environmental rankings can change when materials are compared on a mechanical performance basis [243,244]. This issue is particularly relevant for crashworthy structures because different materials may require different wall thicknesses or masses to provide equivalent structural performance.

Full composite GWP values from cradle-to-gate or equivalent life-cycle assessments were preferentially used when suitable data were available. However, many studies that report SEA do not include corresponding LCA data, and the information needed to reconstruct a complete composite-level GWP is frequently insufficient. Because specimen’s LCAs are generally lacking for the composites examined in crashworthiness research, the present analysis adopts a broader indicator i.e., production-related GWP, as the environmental performance metric. For many NFRC, HFRC, GFRC, and CFRC systems, directly matched whole-composite GWP data were unavailable. In these cases, the composite environmental impact was reconstructed from the reported constituent fractions according to

$$GWP_{comp}=\sum_{i}w_{iGWP_{i}}$$

where *ω_i_* and *GWP_i_* denote the mass fraction and production-related GWP of each constituent, respectively. The constituent GWP values were preferentially derived from cradle-to-gate or factory-gate inventories for fibers and polymer matrices [1,30,245,246].

These reconstructed values represent the environmental burden associated with upstream constituent production. Energy consumption and emissions associated with composite manufacturing processes, including hand lay-up, vacuum assisted resin transfer molding, filament winding, autoclave curing, thermoforming, post-curing, and welding were not added when reliable process level inventories were unavailable. Manufacturing burdens were not estimated using generic assumptions because the energy intensity of composite processing can vary substantially with production route, temperature, cycle time, electricity mix, and production scale [245,246]. Restricting the analysis to composites for which both SEA and complete cradle-to-gate GWP were reported for the exact same formulation would reduce the available dataset substantially and would disproportionately favor conventional materials with more mature life-cycle inventories.

Where GWP ranges were available, the reported range was retained in the database and a representative central value was used for plotting. Mechanical and environmental data from separate sources were matched only when the material composition and production route were considered sufficiently comparable; otherwise, the data point was excluded. Nevertheless, residual uncertainty remains because the source studies differ in loading mode, specimen geometry, strain rate, manufacturing route, electricity mix, allocation procedure, and LCA system boundary. The resulting SEA-GWP map can be used as a comparative engineering screening framework. In particular, small differences among individual points should not be over-interpreted when the underlying mechanical test conditions or environmental accounting boundaries differ.

### 5.3. SEA-GWP Performance Map

**Figure 13:**
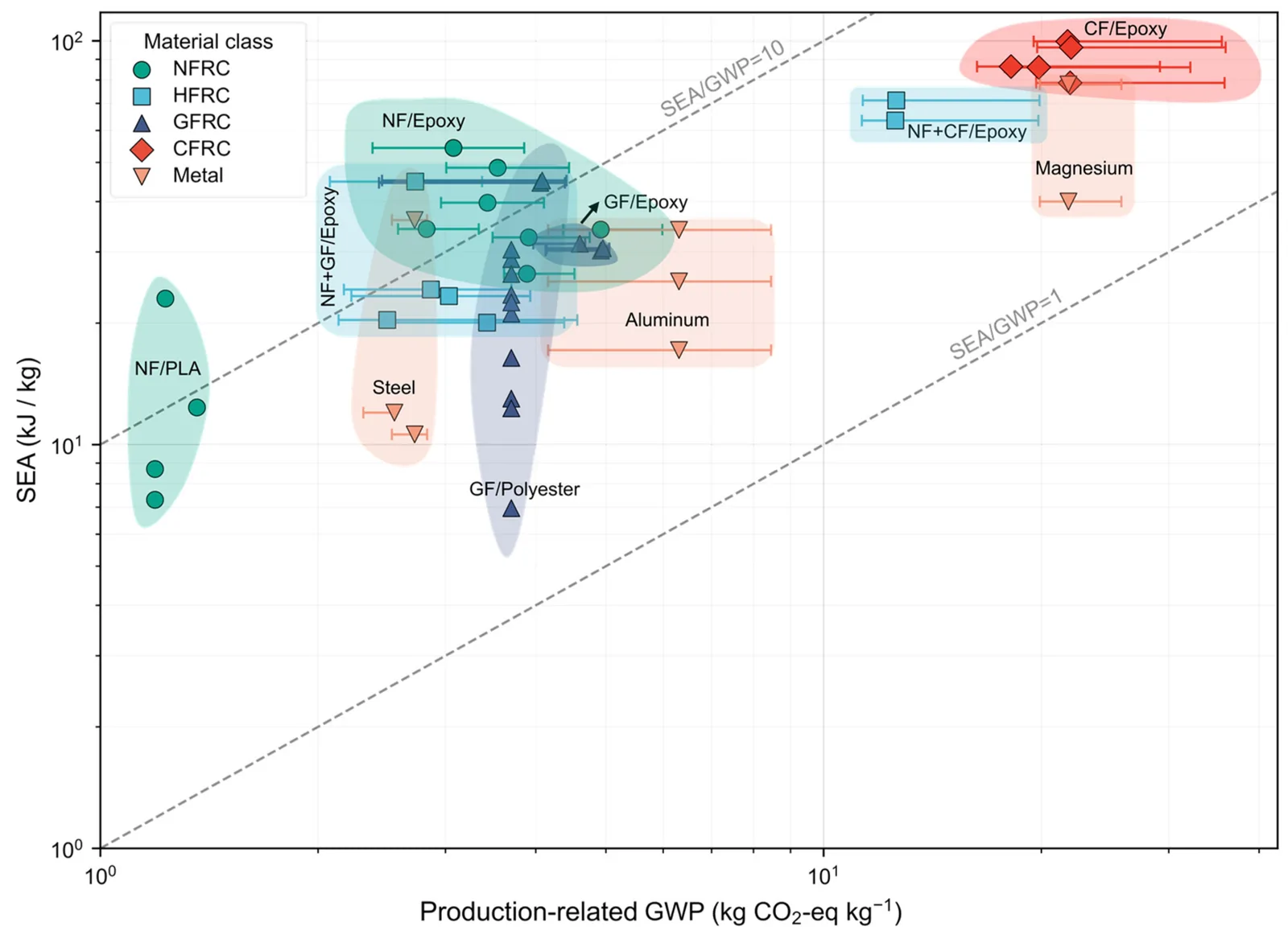
SEA and production-related GWP map of representative energy-absorbing materials [1,6,7,8,9,10,11,12,13,14,15,16,17,18,19,20,21,22,23,24,25,26,27,28,29,30,31,145,247,248,249,250,251,252,253,254,255,256,257,258,259,260].

Figure 13 presents the SEA against production-related GWP for NFRCs, HFRCs, GFRPs, CFRPs, and metallic energy-absorbing systems on logarithmic scales. In this representation, materials located toward the upper-left region are particularly attractive due to their high SEA with relatively low embodied carbon. The dashed lines corresponding to SEA/GWP ratios of 1 and 10 kJ per kg CO_2_-eq provide visual guides for evaluating the combined energy absorption versus carbon performance. These lines could be comparative indicators for identifying material systems that deliver greater energy absorption per unit of greenhouse gas burden.

The map reveals that SEA and GWP do not exhibit a simple proportional relationship. CFRCs occupy the high SEA region but are simultaneously associated with comparatively high production-related GWP. In contrast, most NFRCs are concentrated toward the low-GWP side of the map while maintaining SEA values that overlap substantially with those of GFRPs and metallic absorbers. This distribution demonstrates that increasing the energy-absorption capability does not necessarily require a proportional increase in embodied carbon, and that material architecture, matrix selection, fiber type, and processing route can strongly alter the position of a system within the SEA-GWP design space.

A particularly important feature of the NFRC dataset is its wide SEA range at relatively low GWP. Several epoxy-based NFRCs achieve SEA values of approximately 30–55 kJ/kg, while their reconstructed production-related GWP generally remains below approximately 5 kg CO_2_-eq kg^−1^. Thermoplastic PLA-based systems extend further toward the low-GWP region. For example, the flax/PLA tube included in the present database exhibits a quasi-static SEA of approximately 23 kJ/kg at a reconstructed GWP of approximately 1.23 kg CO_2_-eq kg^−1^, corresponding to an SEA/GWP ratio of 18.7 kJ per kg CO_2_-eq [145]. The matrix is especially important because a high-GWP thermosetting resin can offset a substantial part of the environmental benefit provided by low-GWP natural fibers. This observation indicates that low-carbon thermoplastic matrices can be combined with appropriate fiber architecture and structural designs to achieve favorable energy-absorption performance without the relatively high upstream burden associated with conventional thermosetting matrices.

The SEA/GWP = 10 line provides a particularly useful visual reference for the present dataset. Several NFRC and HFRC systems lie above this line, whereas most CFRPs remain below it despite their higher absolute SEA. This difference highlights the distinction between maximizing energy absorption alone and maximizing energy absorption relative to embodied carbon. The horizontal ranges in Figure 13 show real variation due to different assumptions or processes, not measurement error. For reconstructed composites, they represent sensitivity to constituent GWP values and composition assumptions. For metals, they reflect different production routes or product forms and indicate possible variation from cradle to gate.

### 5.4. Comparison with HFRCs, GFRCs, CFRCs, and Metals

NFRCs offer competitive energy absorption at relatively low production-related GWP, not the highest SEA compared across materials. HFRCs occupy an intermediate region between NFRCs and synthetic-fiber composites. The introduction of glass or carbon reinforcement generally increases both energy-absorption capability and embodied carbon. Several natural/glass hybrid systems in the present dataset achieve SEA values comparable with or higher than those of GFRCs while retaining GWP values substantially below those of CFRCs. Hybridization therefore represents a potentially effective strategy for tuning the compromise between crashworthiness and environmental performance.

The SEA ranges of the GFRCs and NFRCs overlap substantially. Many NFRCs remain positioned further to the left of the SEA-GWP map, indicating lower cradle-to-gate carbon burdens. These results suggest that appropriately designed NFRCs can provide comparable energy absorption performance with reduced embodied carbon. Moreover, GFRPs still have their own strengths, such as well-established production processes and more consistent fiber quality.

A different trade-off is observed for CFRCs. The CFRC systems included in the database exhibit the highest absolute SEA. However, their production-related GWP is also considerably higher in the present dataset. Consequently, their SEA/GWP ratios remain relatively modest compared with several NFRC and HFRC systems. CFRPs remain highly attractive for applications where the main requirement is maximum crash performance per unit mass, such as aerospace and high performance transportation structures. However, when embodied carbon is simultaneously considered as a design objective, the relative attractiveness of NFRCs and HFRCs increases substantially.

Metallic crash absorbers exhibit the broadest distribution in the SEA-GWP design space because their environmental burdens are strongly dependent on alloy type, product form, and production route. Aluminum and magnesium structures can provide relatively high SEA, but their upstream GWP may be substantial, particularly when primary metal production routes are considered. High-strength steels, in contrast, remain highly competitive. This result indicates that the environmental advantage of NFRCs should not be generalized against all metallic systems; instead, comparisons should account for alloy type, recycled content, product form, and production route.

No single material class dominates the entire SEA-GWP design space. CFRCs provide the highest absolute SEA but at relatively high embodied carbon, while GFRCs and HFRCs occupy an intermediate region. High strength steels can also exhibit favorable combined performance. NFRCs are distinguished by their ability to access the low-GWP region while retaining moderate-to-high SEA, particularly when low-carbon matrices and efficient crash architectures are employed. From a sustainable design perspective, the most promising strategy is therefore not simply to replace synthetic fibers with natural fibers but to simultaneously optimize the fiber type, matrix chemistry, reinforcement architecture, structural geometry, and manufacturing route in order to shift energy-absorbing systems toward the upper-left region of the SEA-GWP map.

## 6. Current Progress and Application Readiness of NFRCs in Modern Engineering

NFRCs have advanced from laboratory development toward practical engineering applications, with their technological maturity varying significantly across use cases. Their low density, good specific mechanical properties, damping capacity, renewable feedstocks, and potentially lower environmental impact make them attractive for lightweight engineering [218,261]. However, deployment at industrial scale remains limited by moisture sensitivity, fire performance, long-term durability, property variability, process reproducibility, and qualification requirements, so their readiness should be assessed for each application [218].

Table 4 summarizes the current technological maturity and major barriers of NFRCs in modern engineering applications. The automotive sector is the most mature field for NFRCs. Lignocellulosic-fiber composites have been adopted for interior trim and non-structural lightweight parts, and the focus is now shifting to semi-structural and crash-relevant components. This transition demands strict component-level control of crushing behavior, fiber-interface performance, and manufacturing consistency, as laminate-level energy absorption alone cannot support safety critical structures [218,262]. Aerospace applications are far less mature, restricted to cabin panels and secondary components; stringent fire safety, certification, and durability requirements prevent their use in primary airframe structures [261,263]. In construction, non-structural applications such as insulation panels are verified, but primary load-bearing uses still face major durability challenges in severe service conditions [264]. Railway, marine, and wind energy applications remain mostly at the research stage, constrained by fire standards, degradation, and lack of long-term structural validation [265,266,267].

The main factors that inhibit the widespread use of NFRCs are that the fibers vary considerably, moisture can break down the fiber-matrix bonds, they burn easily, and we still lack sufficient data on how long they last [218]. It is also hard to produce them in large amounts and to set common standards. Methods used to improve performance often add cost and can reduce the green benefits of using plant fibers. Overall, NFRCs can already work in parts that carry little or only some load, but wider use in parts that carry main loads and in safety critical systems will require fixing remaining problems with durability, fire safety, quality control, and certification based on common standards.

Translation of laboratory-scale NFRC energy absorption results to full-scale structures requires staged validation, not direct extrapolation from coupon- or tube-level SEA values. First, material- and coupon-scale tests should establish reliable data on constitutive behavior, damage, strain rate, and environmental degradation, including fiber variability, moisture, temperature, and manufacturing defects, in order to support calibrated models and preliminary screening. Second, representative subcomponents should introduce geometry, wall thickness, joints, triggers, curvature, sandwich architecture, and manufacturing route. The goal is to determine whether progressive crushing or impact damage mechanisms found in laboratory specimens persist when geometry and processing become important. Component scale tests should then add production representative manufacturing, environmental conditioning, repeated loading, joining, and realistic boundary conditions to assess reproducibility and residual performance.

Validation at full scale must be specific to each application since laboratory SEA is an initial screening metric. Automotive structures require crash relevant components such as crush boxes, side impact members, or energy absorbers, followed by subsystem- and vehicle-level crash assessment. Aerospace applications should emphasize secondary or sandwich structures, damage tolerance, fatigue, environmental durability, and fire, smoke, and toxicity qualification before critical use for safety. Protective systems need evaluation of helmet shells, impact panels, and sandwich absorbers under impact sequences, coverage geometry, repeated impact, and post-impact integrity. Across sectors, successful scaling requires correlation between experiments and validated models, statistically defined allowable design, process quality control, durability assessment, inspection criteria, and sector certification. 

## 7. Limitations of Existing Studies

There have been many studies conducted on the effects of impact resistance and energy absorption of NFRCs. However, there are some shortfalls when trying to conduct a quantitative comparison and identify the mechanism of energy absorption, as well as when developing models and applying this knowledge in engineering.

Quantitative comparison and determination of what is best when designing is impossible for various reasons. When producing fibers, the type of fiber used, extraction and processing methods, how much water they contain, fiber volume fraction, how the textile was produced, and how thick the laminate is all play a role. There are also factors such as the matrix used, the process used to create it, porosity, what was used to impact the sample, what boundary conditions were used, and the rate at which it was impacted, all of which can affect the result. Not only are the factors above not linear, they also interact with one another. A higher fiber volume fraction does not always ensure greater energy absorption, too much fiber will cause voids and poor wetting of the fibers. A strong interface prevents fiber pull-out; however, pull-out can absorb a considerable amount of energy. Increasing the wall thickness will not always increase the energy absorbed, it may merely increase the initial peak force required to crush the specimen. All of these examples show that there is an optimum for each material and design. Further studies should be conducted that take all of these factors into account and change one variable at a time or use multivariable tests, allowing for better understanding of the relationship among structure, properties, and energy absorbed.

As mentioned earlier, natural fibers have a lot of variation within them: the diameter, the size and shape of the lumen, how thick the cell walls are, microfibril angles, flaws, and many others. All of these factors will affect the mechanical properties of the fiber. When creating composites, these properties will also vary due to the distribution of fibers, waviness, porosity, etc. Most of the current models used to predict energy absorption act as if the material was homogeneous or equivalent to an orthotropic material. These models can be used to model the force-displacement curve once calibrated, but they cannot reveal how each mechanism interacts and occurs during an impact event. More research needs to be carried out to develop models that can simulate the variation that exists in natural-fiber-reinforced composites. Using multiscale models, probabilistic models, and micro-to-macro homogenization can help with this problem.

There is also a lack of evidence showing each scale at which energy is dissipated during an impact event. Researchers have found matrix cracking, interface failure, fiber pull-out, fiber fracture, delamination, and deformation at the bundle scale, but we do not know how these mechanisms occur or the order in which they take place. We need to further research what is happening at the micro scale and relate it to what we see on a macro scale. By using high-speed cameras, DIC, and data analysis, we can start to understand the deformation that occurs. There are other techniques that can be used, such as acoustic emission and X-ray CT. We can also use microscopy on specimens to observe what is occurring at a smaller scale. Once these tests are conducted, we can use the information to develop more accurate models.

Natural fibers absorb water, so this should be taken into consideration when conducting impact and crushing tests. Many researchers perform these tests in a dry environment. Having a certain amount of moisture in the fiber can increase the energy absorbed, but having too much water over a long period of time can cause the interface to weaken and crack. Research should be carried out on how water affects the lifetime of NFRCs when subjected to impact.

Due to different methods being used to determine SEA and GWP, it is difficult to compare values. Different samples sizes and impact velocities are used to calculate the SEA, while GWP values are calculated by LCAs, which vary in terms of electricity used, components, and whether or not recycling is taken into account. Sometimes, a GWP value is not given for the material used to calculate the SEA. By using the LCA of each component, we can come up with an approximate GWP for our sample. This may not be accurate, but it allows us to better compare materials. In future studies, we should look at improving the accuracy of our GWP values and report energy absorption and life-cycle data using similar methods.

Another reason we cannot apply what we learn from these studies to real world applications is that the samples tested are very small. We need to test these materials at a scale that we would see in cars or other applications. By using models that take into account the variation that occurs in natural-fiber-reinforced composites and validating them with experimental data, we can start to make predictions for larger structures. Additionally, we should look at not only the energy absorbed but also how much force the material can withstand and how much it deforms before failure.

## 8. Conclusions and Future Perspectives

NFRCs have shown great promise as energy absorbers because they are lightweight, made from renewable materials, and can absorb energy through various forms of damage. As discussed in this paper, there are many factors that affect the energy-absorption capability of NFRCs. From fiber to matrix, the interface, fillers, layup configuration, fiber orientation, fiber volume fraction, environment, and structural configuration all play a role in how well an NFRC can absorb energy. On a material level, the matrix will deform and crack, while fibers will break, pull out of the matrix, slide at the interface, and deflect cracks. Depending on the properties of each material, different combinations of these damage modes will occur. Ensuring high strength values for each material will not always yield a higher energy absorption value. It is best to find a balance between damage modes.

Changing the layup configuration and structural configuration can also change the damage modes that occur at the material scale. Changing the fiber orientation, stacking sequence, and fiber volume fraction can greatly affect how these damage modes occur. Some may provide a higher load-carrying capacity, while others may allow for more shear and sliding at the interface, leading to higher energy absorption. As the fiber volume fraction increases, more damage modes will occur, which can lead to higher energy absorption. However, too much fiber can lead to poor impregnation and less matrix deformation, causing lower energy absorption, so there is an optimal fiber volume fraction. Structural configuration also plays a significant role in the damage that occurs when crushing a structure. The shape of the cross-section, wall thickness, D/t ratio, and triggering all affect how the structure will crush. We want to ensure that the structure is breaking up, splaying, folding, and delaminating while avoiding a catastrophic failure or global buckling event, allowing the structure to have a high mean crushing load with a lower initial peak load.

Another factor that can affect the energy absorption of NFRCs is the environment to which they are exposed. As shown above, moisture does not always have a negative effect on the measured energy absorption. If a small amount of moisture is absorbed, it can allow the fibers and matrix to become more ductile, leading to more deformation and sliding at the interface, thereby causing more energy to be absorbed. However, as moisture levels continue to increase, swelling, degradation of the interface, matrix cracking, and delamination will occur, which can lead to lower energy absorption. As seen previously, simply having a high energy-absorption value is not all that matters when evaluating a material’s crashworthiness, factors such as peak load, SEA, CFE, amount of damage, and penetration are also important.

There are still many areas of study that need to be explored when looking at energy absorption in NFRCs. As mentioned earlier, natural fibers are highly heterogeneous, which needs to be taken into consideration when modeling. Many researchers use deterministic properties to represent natural fibers, when in reality they have varying lumen sizes, fiber diameters, and many other characteristics that need to be taken into account. By using micromechanics, stochastic modeling, and cohesive zone modeling, we can better understand how these characteristics affect a structure’s crashworthiness.

Along with modeling, we need to perform tests that can show us what damage is occurring during impact and crushing. Using techniques such as high-speed cameras, DIC, acoustic emission, X-ray CT, and in situ microscopy, we can start to understand what damage modes occur first and how they interact with one another. This information can then be used to build and improve upon our current multiscale models. Once we know how each damage mode is contributing to the overall energy absorption, we can use data-driven methods to find optimal material combinations. As shown above, most research is being carried out on samples or tubes specimens at laboratory scale. Further research should be conducted on component level structures and account for environmental conditions. Once we are able to correlate all of these things together, we can start to find the right balance of energy absorption, aiming to find not only a material that can absorb a lot of energy but one that has a lower peak load and can retain its properties after impact. By understanding the relationships among material, damage, and structure, we can begin to use NFRCs as energy absorbing components in engineering applications.

## Figures and Tables

**Table 1:** Summary of experimental methods used to evaluate the energy-absorption behavior of NFRCs (data were sourced from Refs. [42,43,44,45,46,47,48,49,50,51,52,53,54,55,56,57,58,59,60,61,62,63,64,65,66]).

Test Method	Main Methodology and Loading Conditions	Key Outputs	Standards/Methodological Basis
Quasi-static compression	Uniaxial compression under displacement control	Stress-strain response, compressive strength, damage mode	ISO 14126; ASTM D6641
Quasi-static axial crushing	Axial loading between rigid platens; 0.1–10 mm/min	EA, SEA, MCF, PCF, CFE, collapse mode	Study-specific crashworthiness configurations
Drop-weight impact	Instrumented impactor with prescribed mass/velocity/incident energy	Peak force, displacement, absorbed energy, damage area, rebound/perforation behavior	Study-specific impact configurations in the cited literature
SHPB compression	Incident/reflected/transmitted stress waves; dynamic stress equilibrium and controlled strain rate required	Dynamic stress-strain response, peak stress, strain-rate sensitivity, absorbed energy, failure mode	One-dimensional stress-wave theory; pulse-shaping and equilibrium criteria

**Table 2:** Quantitative evidence linking interface-controlled damage mechanisms to energy absorption in NFRCs.

System/Modification	Quantitative Response	Dominant Damage Change	Mechanistic Implication
Flax/epoxy + cellulose acetate interphase	Perforation threshold energy +66%; perforation energy +42%	More fiber–matrix debonding; more ductile failure	Controlled interfacial debonding can improve impact tolerance when load transfer remains sufficient.
GO-modified flax/epoxy	Interfacial adhesion +40%; void content < 0.04%; perforation energy ~unchanged	More pull-out and interfacial sliding; delayed damage initiation	Stronger adhesion can redistribute dissipation mechanisms without necessarily increasing the total absorbed energy.
Flax/epoxy/GO	Crack initiation energy ~1.6 times; crack propagation energy ~0.79 times	Delayed initiation; reduced propagation-related damage	Interface modification shifts the energy balance from damage propagation toward damage initiation.
Flax/MAPP vs. flax/epoxy	Perforation energy > 50% higher; Impact damaged area 38–59% lower	Limited delamination; greater matrix/plastic dissipation	High total energy absorption does not require extensive delamination.

**Table 3:** Effects of interfacial modification and particulate fillers on the energy-absorption behavior of NFRCs.

Modification Approach	Representative Systems	Effect on Energy Absorption	Main Mechanisms and Limitations
Alkali/silane surface treatment [148,149,150,151,152]	NaOH- and/or silane-treated natural-fiber composites	Moderate treatment can improve damage resistance, but total absorbed energy does not necessarily increase.	Improved roughness and interfacial adhesion enhance load transfer; excessive treatment may damage fibers or suppress debonding and pull-out, reducing frictional dissipation.
Interfacial toughening/modification [126,127]	Cellulose-acetate or GO-modified flax composites	Promotes more progressive damage and improved damage tolerance; perforation energy increased by up to 42% in some systems.	Reduces stress concentration and promotes controlled debonding, sliding, and distributed damage; excessive interfacial strengthening may reduce beneficial dissipation mechanisms.
Natural/biomass particulate fillers [153,154,155,156,157,158]	Peanut-shell powder, wood powder, biochar, shell particles	Low-to-moderate filler contents can improve impact performance, whereas excessive loading generally reduces it.	Crack pinning/deflection, particle debonding, friction, and matrix deformation increase dissipation; agglomeration, voids, and poor wetting dominate at high contents.
Micro-/nanoscale fillers [159,160]	TiO_2_- and SiO_2_-modified natural-fiber composites	Appropriate nanoparticle contents can markedly improve impact-energy absorption; further addition is often detrimental.	Nanoparticles hinder crack propagation and increase fracture-path tortuosity; excessive loading increases viscosity, agglomeration, and porosity and may embrittle the matrix.

**Table 4:** Current technological maturity and major barriers of NFRCs in modern engineering applications [218,261,262,263,264,265,266,267].

Sector	Representative Applications	Current Maturity	Main Advantages	Major Barriers
Automotive	Interior trim, door panels, headliners, seats; crash parts	Commercial for interiors; developing for semi-structural use	Low density, damping, formability, thermoplastic processing	Property scatter, moisture, crash reproducibility, joining, fatigue, component-scale validation
Aerospace	Cabin panels, sandwich structures, secondary components	Low maturity for primary structures	Lightweighting, damping, less synthetic fiber	Fire/smoke, moisture, fatigue, damage tolerance, certification
Construction	Insulation, acoustic panels, cementitious/polymer composites, strengthening systems	Mature for non-structural use; developing for structural use	Renewable, low density, insulation, lower embodied impact	Moisture, alkaline degradation, biological durability, fire, creep, standards
Railway	Interior and possible semi-structural components	Early/limited adoption vs. synthetic composites	Weight reduction, damping, renewable reinforcement	Fire regulations, durability, mechanical consistency, production scale, certification
Marine	Interior panels, decks, sandwich structures, small craft/secondary parts	Limited applications	Low density, corrosion resistance, renewable content	Water uptake, swelling, interfacial degradation, long-term marine durability
Wind energy	Blade materials, substructures, natural/synthetic hybrid blades	Research/demonstration stage	Low density, renewable reinforcement, possible environmental/end-of-life benefits	Fatigue, stiffness retention, moisture, joining, long service life, blade-scale validation
Energy absorption	Crash absorbers, impact panels, helmets, sandwich structures	Laboratory/prototype stage	Progressive damage, specific energy dissipation, damping, low mass	Rate dependence, reproducibility, environmental stability, manufacturing consistency, certification

## Data Availability

No new data were created or analyzed in this study. Data sharing is not applicable to this article.

## Abbreviations

The following abbreviations are used in this manuscript:

NFRCs	Natural fiber reinforced composites
LCA	Life-cycle assessments
UNSDGs	United Nations Sustainable Development Goals
SDG	Sustainable Development Goal
CFRCs	Carbon-fiber-reinforced composites
GFRCs	Glass-fiber-reinforced composites
SEA	Specific energy absorption
GWP	Global warming potential
GWPf	Global warming potential of reinforcing fibers
AFRCs	Aramid-fiber-reinforced composites
CO_2_	Carbon dioxide
MAPP	Maleic anhydride polypropylene
PLA	Polylactic acid/poly(lactic acid)
HDPE	High-density polyethylene
SHPB	Split Hopkinson Pressure Bar
EA	Energy absorption/absorbed energy
MCF	Maximum crushing force
PCF	Plateau crushing force
CFE	Crushing efficiency/crushing force efficiency
ISO	International Organization for Standardization
ASTM	American Society for Testing and Materials
GO	Graphene oxide
NaOH	Sodium hydroxide
SEM	Scanning electron microscopy
CGFs	Cryptostegia grandiflora fibers
UV	Ultraviolet
TiO_2_	Titanium dioxide
SiO_2_	Silicon dioxide
PA6	Polyamide 6
PP	Polypropylene
UD	Unidirectional
2D	Two-dimensional
3D	Three-dimensional
HFRCs	Hybrid fiber reinforced composite materials
wt.%	Weight percent
vol.%	Volume percent
D/t	Diameter-to-thickness ratio
